# Neutrophil-mediated innate immune resistance to bacterial pneumonia is dependent on Tet2 function

**DOI:** 10.1172/JCI171002

**Published:** 2024-04-04

**Authors:** Candice Quin, Erica N. DeJong, Elina K. Cook, Yi Zhen Luo, Caitlyn Vlasschaert, Sanathan Sadh, Amy J.M. McNaughton, Marco M. Buttigieg, Jessica A. Breznik, Allison E. Kennedy, Kevin Zhao, Jeffrey Mewburn, Kimberly J. Dunham-Snary, Charles C.T. Hindmarch, Alexander G. Bick, Stephen L. Archer, Michael J. Rauh, Dawn M.E. Bowdish

**Affiliations:** 1 Department of Medicine, Faculty of Health Sciences, McMaster University, Hamilton, Ontario, Canada.; 2 Firestone Institute for Respiratory Health, St. Joseph’s Healthcare, Hamilton, Ontario, Canada.; 3 Institute of Medical Sciences, School of Medicine, Medical Sciences and Nutrition, University of Aberdeen, Aberdeen, United Kingdom.; 4 Department of Pathology and Molecular Medicine, Faculty of Health Sciences,; 5 Department of Medicine,; 6 Department of Biomedical and Molecular Sciences, and; 7 Queen’s CardioPulmonary Unit, Queen’s University, Kingston, Ontario, Canada.; 8 Division of Genetic Medicine, Department of Medicine, Vanderbilt University Medical Center, Nashville, Tennessee, USA.

**Keywords:** Immunology, Infectious disease, Innate immunity

## Abstract

Individuals with clonal hematopoiesis of indeterminate potential (CHIP) are at increased risk of aging related health conditions and all-cause mortality, but whether CHIP affects risk of infection is much less clear. Using UK Biobank data, we revealed a positive association between CHIP and incident pneumonia in 438,421 individuals. We show that inflammation enhanced pneumonia risk, as CHIP carriers with a hypomorphic IL6 receptor polymorphism were protected. To better characterize the pathways of susceptibility, we challenged hematopoietic *Tet Methylcytosine Dioxygenase 2–*knockout (*Tet2^–/–^*) and floxed control mice (*Tet2^fl/fl^*) with *Streptococcus pneumoniae*. As with human CHIP carriers, *Tet2^–/–^* mice had hematopoietic abnormalities resulting in the expansion of inflammatory monocytes and neutrophils in peripheral blood. Yet, these cells were insufficient in defending against *S*. *pneumoniae* and resulted in increased pathology, impaired bacterial clearance, and higher mortality in *Tet2^–/–^* mice. We delineated the transcriptional landscape of *Tet2^–/–^* neutrophils and found that, while inflammation-related pathways were upregulated in *Tet2^–/–^* neutrophils, migration and motility pathways were compromised. Using live-imaging techniques, we demonstrated impairments in motility, pathogen uptake, and neutrophil extracellular trap (NET) formation by *Tet2^–/–^* neutrophils. Collectively, we show that CHIP is a risk factor for bacterial pneumonia related to innate immune impairments.

## Abstract 2

Individuals with TET2-mutant clonal hematopoiesis of indeterminate potential are at increased risk of bacterial pneumonia due to innate immune impairments.

## Introduction

Human aging is accompanied by dysregulation of hematopoiesis in the bone marrow (BM), which may have adverse clinical consequences (1). Hematopoiesis is a tightly regulated process by which hematopoietic stem cells (HSC) differentiate into functional and mature blood cells. As HSCs reside and cycle in the BM, they naturally acquire mutations that are then passed on to their progeny, resulting in clonal hematopoiesis. Acquired mutations that are advantageous to cell survival accumulate over time (2). When the resultant mutant clones and their variant alleles are found at a frequency of 2% or more in peripheral blood cell DNA, this is defined as clonal hematopoiesis of indeterminate potential (CHIP) (3). The occurrence of CHIP increases with age and it is estimated that 10%–20% of adults aged 65 and above are CHIP carriers (4). By contrast, somatic CHIP clones are detectable in less than 1% of individuals under the age of 40 (5). The mutational events that drive CHIP occur most frequently in the *DNA Methyltransferase 3* α (*DNMT3A*)*,* and *Tet Methylcytosine Dioxygenase 2* (*TET2*) genes (6). Although these genes have seemingly opposing enzymatic roles on DNA methylation, *DNMT3A* methylating versus *TET2* demethylating (7), many studies have demonstrated convergent effects of mutations in these genes on inflammatory and hematopoietic pathways. For instance, both *TET2* and *DNMT3A* inactivating mutations appear to have important roles in dysregulating the differentiation and function of myeloid cells like monocytes and neutrophils. Both human and murine studies have shown that loss of function in either *TET2* (8–10) or *DNMT3A* (11) biases hematopoiesis toward the myeloid lineage, which ultimately results in an imbalanced ratio of myeloid/lymphoid cells, termed myeloid skewing. Monocytes isolated from mutant-*DNMT3A* carriers have increased expression of proinflammatory genes compared with those without *DNMT3A* mutations (12). This includes genes encoding inflammatory mediators such as IL6 and IL8, chemokine (C-C motif) ligands 4 (CCL4) and resistin, which promotes monocyte adhesion to endothelial cells (12, 13). Several studies have also supported a role for mutant-*TET2* clones in promoting macrophage inflammation and the secretion of proinflammatory mediators, including IL6 (14–16). It has been suggested that aberrant inflammation from CHIP myeloid clones may underpin various comorbid diseases such as hematopoietic cancers and cardiovascular disease (17, 18), including pulmonary arterial hypertension (19). More recently, a relationship between clonal hematopoiesis and risk of severe infections, including various lung infections, has been identified (20), but the mechanisms and pathways of susceptibility are not known.

Lower respiratory tract infections such as pneumonia are among the most common cause of infectious disease–related hospitalizations and are a leading cause of morbidity and mortality worldwide (21). Older adults are at a higher risk of developing pneumonia and having more adverse outcomes (22). This increased risk is thought to be driven by an age-related remodeling of the immune system (1). With increasing age, HSCs committed to the myeloid lineage outnumber those committed to the lymphoid lineage in both humans and mice, resulting in myeloid skewing (23). This phenomenon is accompanied by age-related changes in myeloid cell subsets that favor inflammation. For instance, circulating Ly6C^hi^ inflammatory monocytes increase with age in mice and express more of the chemokine receptor CCR2 (24). These monocytes — which are equivalent to CD14^+^CD16^–^ classical and CD14^+^CD16^++^ inflammatory monocytes in humans (25) — produce higher levels of proinflammatory cytokines than their Ly6C^lo^/nonclassical counterparts, and, as a result, are often associated with immunopathology (24, 26). Moreover, in humans, having higher than age-average levels of inflammatory cytokines in circulation increases the risk of developing pneumonia and other age-related diseases (27). Although the link between age-associated inflammation and poor clinical outcomes to respiratory infections has a robust epidemiological basis, it lacks conclusive mechanistic explanations (28). We and others (20, 29) postulate that clonal hematopoiesis, which connects aging, aberrant myelopoiesis and inflammation, may explain this phenomenon.

To determine whether CHIP has a disease-modifying role in pneumonia, we leveraged data from the UK Biobank to establish a relationship between CHIP and incident pneumonia. We then challenged *Tet2* hematopoietic knockout (*Tet2^–/–^*) and floxed control mice (*Tet2^fl/fl^*) with *Streptococcus pneumoniae* to better characterize the pathways of susceptibility in mutant-*TET2* CHIP. The research presented here suggests that naturally occurring mutations in *TET2* are a major risk factor for bacterial pneumonia, driven by myeloid immune cell dysfunction. Critically, this work highlights dysfunctional *Tet2*-mutant neutrophils as pivotal cells in *S*. *pneumoniae* pathogenesis and a possible target for therapeutic intervention in CHIP carriers.

## Results

### CHIP is a risk factor for bacterial pneumonia.

**Figure 1:**
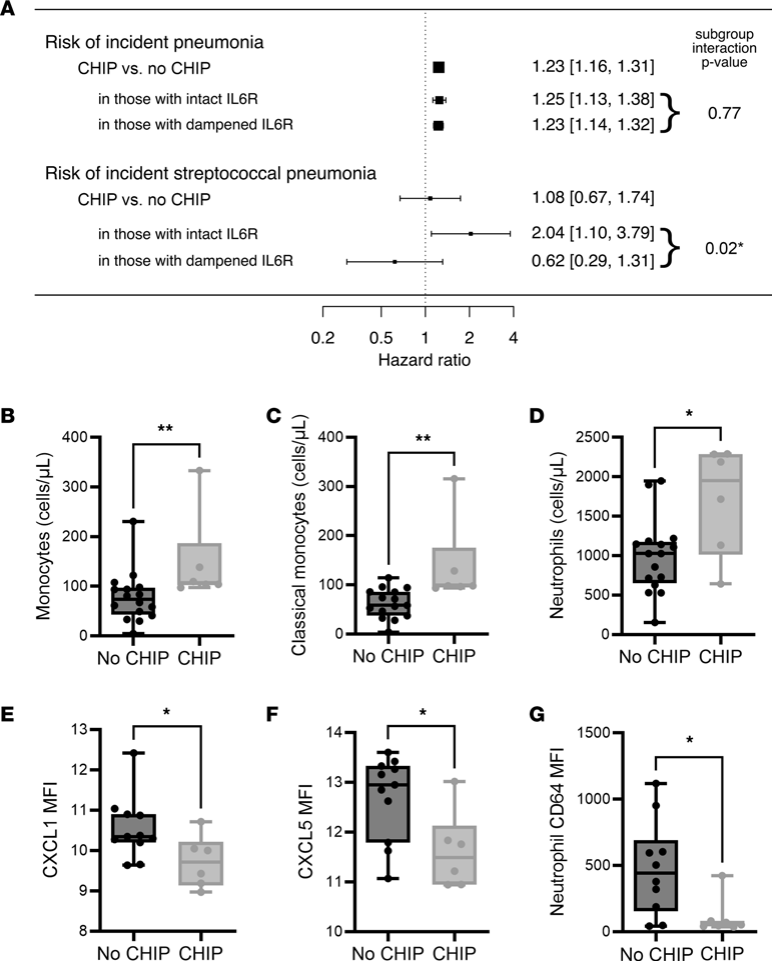
CHIP is positively associated with incident pneumonia caused by *Streptococcus pneumoniae*. (**A**) CHIP is positively associated with incident all-cause pneumonia among 438,421 individuals in the UK Biobank without a history of pneumonia in a Cox proportional hazards regression model adjusted for age, age^2^, sex, smoking history, history of chronic inflammatory lung disease (COPD), and 10 principal components of genetic ancestry. In a model adding an interaction term for a common SNP in the IL6 receptor associated with lower IL6 signaling (rs2228415), CHIP is associated with incident confirmed *S*. *pneumoniae* pneumonia. The CHIP × rs2228415 interaction term is significantly below 0, suggesting that lower IL6R signaling mitigates the effects of CHIP on pneumonia risk. (**B**–**G**) Leukocyte populations were quantified in whole blood from CHIP carriers and noncarriers in a local cohort using flow cytometry. Compared with noncarriers, the numbers of (**B**) peripheral blood monocytes (No CHIP [78.4 ± 12.9]; CHIP [147.6 ± 37.5]), (**C**) classical monocytes (No CHIP [62.4 ± 7.7]; CHIP [138.3 ± 35.9]), and (**D**) neutrophils (No CHIP [990 ± 117]; CHIP [1709 ± 281]), were increased in CHIP carriers. Chemokines (**E**) CXCL1 (No CHIP [10.5 ± 0.23]; CHIP [9.7 ± 0.26]), and (**F**) CXCL5 (No CHIP [12.7 ± 0.25]; CHIP [11.6 ± 0.32]), were decreased in the sera of CHIP carriers. (**G**) Surface expression of CD64 was decreased on circulating blood neutrophils in CHIP carriers (No CHIP [474.9 ± 113.5]; CHIP [106.6 ± 53.0]). Data are presented as box and whisker plots, minimum to maximum, where the center line represents the median and each dot is a participant. Sample size: 16 No CHIP, 6 CHIP participants. Significant outliers removed using ROUT method. MFI, Geometric Mean Fluorescence Intensity. Significance was assessed by Mann-Whitney test. *P ≤ 0.05, **P ≤ 0.01.

We sought to determine the role of CHIP in pneumococcal disease risk using data from the UK Biobank. The cohort included 438,421 individuals, of whom 14,787 (3.3%) had CHIP clones detected at variant allele fraction (VAF) of at least 2%. In agreement with previous reports (30), mutations in *TET2* and *DNMT3A* were the most frequently identified mutated genes associated with CHIP. We demonstrate that risk of pneumonia events was increased in individuals with CHIP (Figure 1A). CHIP carriers had a 1.23-fold higher risk of incident pneumonia than noncarriers after correcting for age, sex, and other covariates. These epidemiological data indicate that there is a significant association between CHIP carrier status and incidence of pneumonia.

Previous reports have demonstrated that inflammatory mediators such as IL6 increase CHIP-comorbid risk to cardiovascular disease (31). To determine if inflammation influenced the risk of incident pneumonia in CHIP carriers, we added an interaction term for a common SNPs in the IL6 receptor gene (*IL6R* [rs2228415]), which reduces IL6 signaling. We show that a heightened risk of *Streptococcus pneumoniae* pneumonia in CHIP (Figure 1A) carriers was abrogated in individuals with this hypomorphic IL6R SNP (in those with low genetic IL6R). These results indicate that a lower inflammatory status may be beneficial for reducing pneumonia risk in CHIP carriers.

### CHIP contributes to myeloid expansion.

Abnormal peripheral blood neutrophil and monocyte numbers may increase pneumonia risk (32). Using flow cytometry, we examined peripheral blood leukocytes in 22 participants from the greater Hamilton area in Canada with or without CHIP at a steady state, ascertained using our previously described 48-gene targeted CHIP panel (33). The characteristics of the study population are displayed in Supplemental Table 1 (supplemental material available online with this article; https://doi.org/10.1172/JCI171002DS1 The participants ranged from 56 to 100 years of age and consisted of 14 females (63.6%) and 8 males. There were no significant differences in BMI or sex distribution among participants. Of the CHIP carriers (*n* = 6), 50% had mutant-*DNMT3A* clones and 50% had mutant-*TET2* clones detected as variant allele fraction (VAF) at 2% or more. We found that innate immune cell numbers varied significantly with CHIP carrier status such that CHIP carriers had higher numbers of total monocytes, classical monocytes, and neutrophils (Figure 1, B–D) in whole blood, consistent with myeloid expansion. No differences in absolute and relative numbers of intermediate and nonclassical monocytes were observed. We found that the CHIP carriers had lower levels of the chemokines CXCL1 and CXCL5 in serum, which are important for neutrophil recruitment and mobilization in response to lung infections (Figure 1, E and F). CHIP carriers also expressed lower levels of the high-affinity Fc-γ receptor, CD64, on circulating neutrophils (Figure 1G). Taken together, these data provide evidence that alterations in myeloid cell populations in CHIP carriers may increase risk of bacterial pneumonia.

### Tet2 mutations increase innate immune cell numbers and inflammation in circulation.

**Figure 2:**
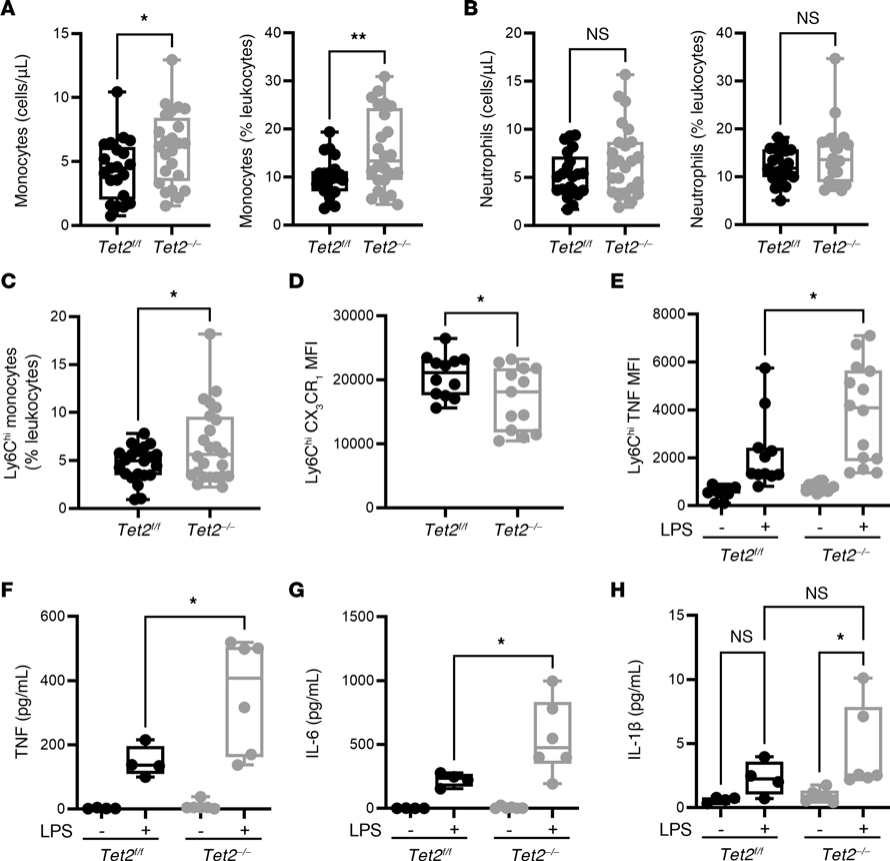
Expansion of inflammatory monocytes in peripheral blood following *TET2* loss. (**A**) Flow cytometric analysis of peripheral blood revealed an increase in the number (*Tet2^fl/fl^* [111 ± 13.07], *n* = 21; *Tet2^–/–^* [157.5 ± 15], *n* = 24) and proportion (*Tet2^fl/fl^* [9.85 ± 0.89]; *Tet2^–/–^* [15.43 ± 1.66]) of monocytes in *Tet2^–/–^* mice. (**B**) There was a tendency toward increase in the number (*Tet2^fl/fl^* [135 ± 12.7], *n* = 21; *Tet2^–/–^* [177.4 ± 18.58], *n* = 24) and relative proportion (*Tet2^fl/fl^* [12.3 ± 0.78]; *Tet2^–/–^* [14.4 ± 1.34]) of circulating neutrophils in *Tet2^–/–^* mice. (**C**) The proportion of Ly6C^hi^ inflammatory monocytes, as a proportion of total CD45^+^ leukocytes, increased in the circulation of *Tet2^–/–^* mice (6.63 ± 0.81) compared with *Tet2^fl/fl^* mice (4.60 ± 0.39). (**D**) This corresponded with a decrease in the surface expression of CX_3_CR_1_ (*Tet2^fl/fl^* [20,657 ± 945.6]; *Tet2^–/–^* [17,057 ± 1355]). (**E**) Intracellular staining of TNF revealed higher TNF expression in the peripheral blood of *Tet2^–/–^* mice (4,050 ± 662) following 4-hour stimulation with LPS, compared with *Tet2^fl/fl^* mice (2,202 ± 566.4) (**F**) Results from a multiplex-ELISA showed that whole blood from *Tet2^–/–^* mice had a significant increase in TNF (*Tet2^fl/fl^* [147 ± 24.3]; *Tet2^–/–^* [357 ± 71.2]), and (**G**) IL6 (*Tet2^fl/fl^* [225 ± 26.3]; *Tet2^–/–^* [553 ± 119]) compared with *Tet2^fl/fl^* mice, following 4 hour stimulation with LPS ex vivo. (**H**) There was a significant induction of IL1-β in whole blood from *Tet2^–/–^* mice following LPS stimulation (4.48 ± 1.3) that was not observed in blood stimulated from *Tet2^fl/fl^* mice (2.3 ± 0.7). Data are presented as box and whisker plots, minimum to maximum, where the center line represents the median and each dot is a mouse. MFI, Geometric Mean Fluorescence Intensity. Significance was assessed by Kruskal-Wallis test. *P ≤ 0.05, **P ≤ 0.01.

To better characterize the pathways of susceptibility, we employed mice with *Tet2*-KO directed to the hematopoietic system (*Vav1-iCre^+^;Tet2^–/–^*, i.e., *Tet2^−/−^*) and control (*Vav1-iCre-;Tet2^fl/fl^*, i.e., *Tet2^fl/fl^*) mice. As with human CHIP carriers, the *Tet2^–/–^* mice had hematopoietic abnormalities at a steady state, resulting in the expansion of both the number and relative proportion (as a percentage of CD45^+^ leukocytes) of monocytes in peripheral blood (Figure 2A). Though not significant, there was also a tendency for blood neutrophil counts to increase (Figure 2B). Murine monocytes can be divided into subsets by their surface expression of the glycoprotein Ly6C into Ly6C^lo^ and Ly6C^hi^ monocytes (34, 35). Monocytes expressing high levels of Ly6C have proinflammatory functions and tend to express low levels of CX3C chemokine receptor 1 (CX_3_CR_1_) (25). Accordingly, we found that the proportion of Ly6C^hi^ monocytes was higher in *Tet2^–/–^* mice, while the proportion expressing low CX_3_CR_1_ was decreased (Figure 2, C and D). Consistent with their ascribed function, we demonstrated that these monocytes were more inflammatory and produced higher levels of tumor necrosis factor α (TNF) in response to LPS stimulation (Figure 2E). Subsequent analyses using a multiplexed-ELISA for key proinflammatory cytokines (TNF, IL6, and IL1-β) reaffirmed the exacerbated TNF response in the serum of *Tet2^–/–^* mice following stimulation with LPS (Figure 2F). Additionally, we found an increased IL6 response in *Tet2^–/–^* mice compared with *Tet2^fl/fl^* mice (Figure 2G) and a significant induction of IL1-β that was not observed in the *Tet2^fl/fl^* mice following LPS stimulation (Figure 2H). Taken together, these data demonstrate that changes in monocyte subsets may contribute to excessive inflammation in *Tet2^–/–^* mice during bacterial challenge.

### Tet2 regulates expansion and emigration of myeloid cell lineages.

**Figure 3:**
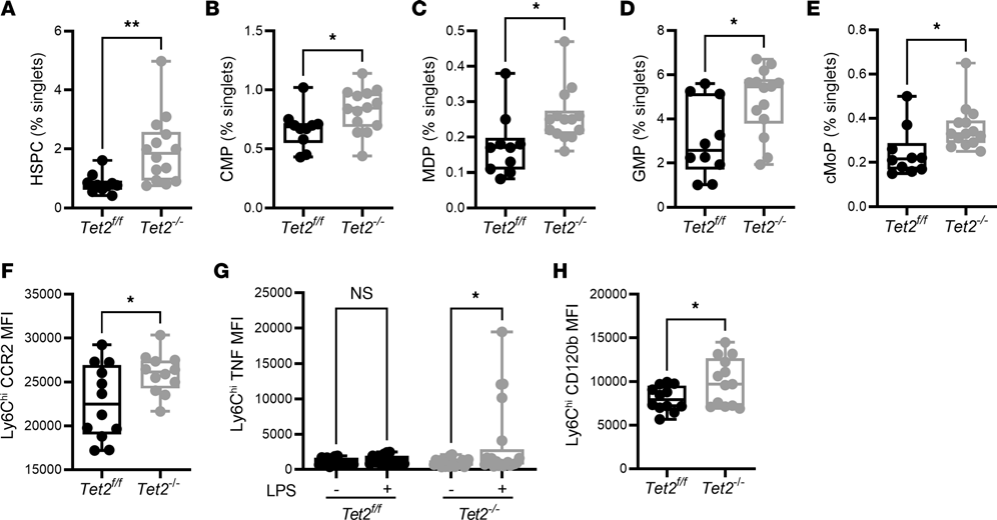
Mutations in *Tet2* increase the proportion of myeloid progenitor cells in the bone marrow. Flow cytometry analysis of the hematopoietic compartment showed an increase in the relative proportion of (**A**) hematopoietic stem and progenitor cells (HSPC) (*Tet2^fl/fl^* [0.83 ± 0.10], *n* = 10; *Tet2^–/–^* [1.95 ± 0.30], *n* = 14), (**B**) common myeloid progenitor (CMP) (*Tet2^fl/fl^* [0.66 ± 0.05]; *Tet2^–/–^* [0.82 ± 0.04]), (**C**) monocyte-dendritic progenitor (MDP) (*Tet2^fl/fl^* [0.17 ± 0.02]; *Tet2^–/–^* [0.25 ± 0.02]), (**D**) granulocyte-monocyte progenitors (GMP) (*Tet2^fl/fl^* [3.06 ± 0.54]; *Tet2^–/–^* [4.82 ± 0.40]), (**E**) and common monocyte progenitors (cMoP) (*Tet2^fl/fl^* [0.24 ± 0.03]; *Tet2^–/–^* [0.35 ± 0.02]) in *Tet2^–/–^* mice compared with *Tet2^fl/fl^* mice. (**F**) Inflammatory Ly6C^hi^ monocytes within the bone marrow of *Tet2^–/–^* mice had higher expression of the surface C-C chemokine receptor type 2 (CCR2) (*Tet2^fl/fl^* [22695 ± 1222]; *Tet2^–/–^* [25971 ± 656]), compared with *Tet2^fl/fl^* mice. (**G**) These monocytes were hyper-responsive to ex vivo stimulation with LPS and had a significant induction of intracellular TNF expression, whereas monocytes from *Tet2^fl/fl^* did not. (**H**) Expression of the cell surface TNF receptor, CD120b, was increased on mutant-*TET2* Ly6C^hi^ monocytes (*Tet2^fl/fl^* [8055 ± 412]; *Tet2^–/–^* [10013 ± 746]) in the bone marrow. Data are presented as box and whisker plots, minimum to maximum, where the center line represents the median and each dot is a mouse. MFI, Geometric Mean Fluorescence Intensity. Significance was assessed by Mann-Whitney test. *P ≤ 0.05, **P ≤ 0.01.

To determine whether *Tet2-*related changes in monocyte numbers, phenotype, and inflammatory capacity were related to changes in myelopoiesis, we examined hematopoietic stem and progenitor (HSPC) cell populations in the BM. In agreement with previous reports (8), we found that *Tet2* loss-of-function increased HSPC proliferation, giving rise to an increase in myeloid-biased multipotent progenitor cells. We found that the proportion of common myeloid progenitors (CMP), monocyte-dendritic progenitors (MDP), granulocyte-monocyte progenitors (GMPs), and common monocyte progenitors (cMoP) all increased in *Tet2^–/–^* mice (Figure 3, A–E). Since progenitor numbers were similar between the groups, we sought to determine if an increase in monocyte emigration in *Tet2^–/–^* mice could account for differences in circulating myeloid populations. The C-C chemokine receptor type 2 (CCR2) is required for leukocytes, and especially Ly6C^hi^ monocytes to leave the BM and enter the blood (36). As such, we hypothesized that enhanced CCR2 expression could prompt their emigration from the BM and could further explain their increased number seen in the circulation of *Tet2^–/–^* mice. In support of this notion, we demonstrated a significant increase in Ly6C^hi^ CCR2 expression in the BM of *Tet2^–/–^* mice (Figure 3F). Thus, mobilization of inflammatory monocytes to the blood may be increased in *Tet2^–/–^* mice.

We have previously shown that *Tet2-*deficient BM progenitors have a proliferative advantage in the presence of TNF (37). Based on this, we explored whether differences in bone marrow TNF responsiveness exist between *Tet2^–/–^* and *Tet2^fl/fl^* mice. We found, as in the peripheral blood, that inflammatory Ly6C^hi^ monocytes in BM express higher TNF following stimulation with LPS (Figure 3G). Not only was TNF increased, but the expression of the TNF receptor CD120b (TNFR2) was as well (Figure 3H). Taken together, these data suggest that an inflammatory environment may propagate the expansion of *TET2-*mutant myeloid cells, contributing to clonal dominance.

### Tet2^–/–^ mice exhibit a pathological myeloid response to S. pneumoniae.

**Figure 4:**
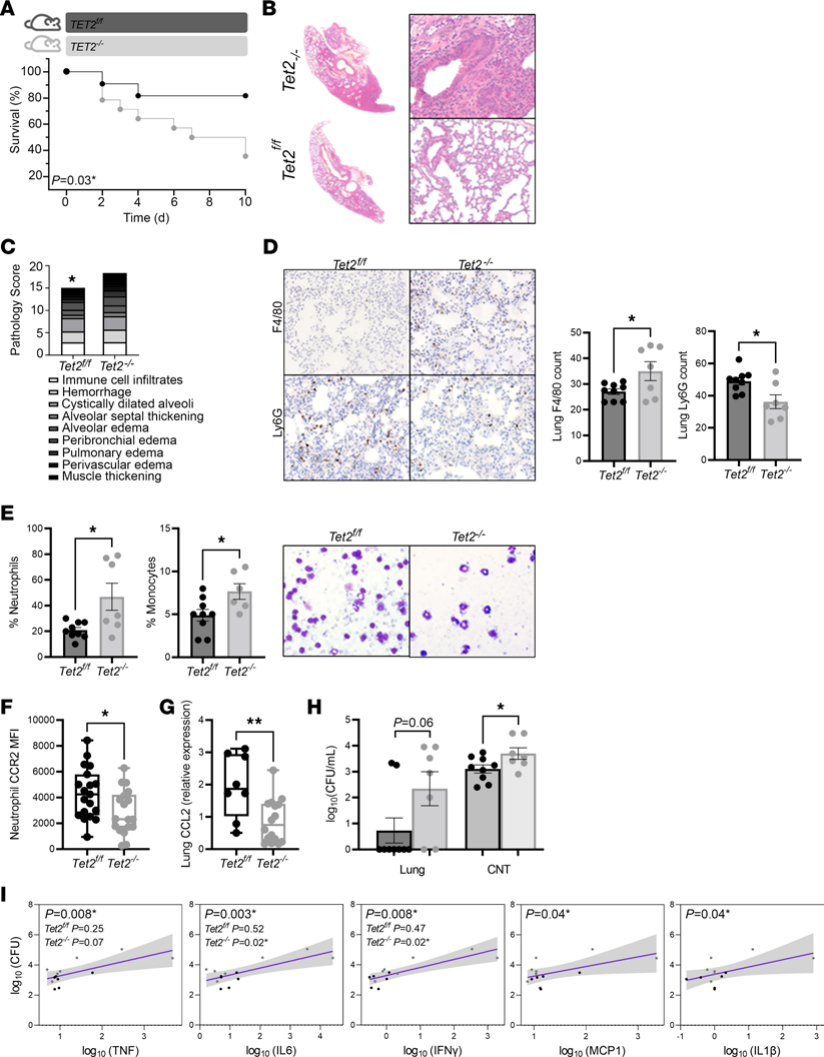
Pneumococcal pneumonia–induced sepsis and accompanying inflammatory responses are exacerbated in Tet2^–/–^ mice. (**A**) Experimental timeline of infection and concurrent survival (*n* = 13 *Tet2^–/–^*; *n* = 12 *Tet2^fl/fl^*). (**B**) Representative H&E-stained lung sections of mice at 10 days p.i. with *S*. *pneumoniae*. Original magnification 20-fold. (**C**) Histopathological analysis of lung H&E tissue sections [as shown in (**B**)] attained by 2 blinded scorers. (**D**) Counts and representative IHC staining of mononuclear phagocytes (F4/80^+^) and neutrophils (Ly6G^+^) on lung sections at 10 days p.i. Original magnification 200-fold. *Tet^–/–^* mice had increased numbers of mononuclear phagocytes in the lungs (*Tet2^fl/fl^* [27 ± 1]; *Tet2^–/–^* [35 ± 3.6]). In contrast, neutrophils were decreased (*Tet2^fl/fl^* [49 ± 2.3]; *Tet2^–/–^* [36 ± 4.2]). (**E**) Relative frequency, as a percentage of 100 cells counted via LeukoSpins, of neutrophils and monocytes in circulation 10 days p.i. showed an increase in neutrophils (*Tet2^fl/fl^* [20.9 ± 2.1]; *Tet2^–/–^* [46.9 ± 10.5]) and monocytes (*Tet2^fl/fl^* [4.9 ± 0.7]; *Tet2^–/–^* [7.6 ± 2.2]) in *Tet2^–/–^* mice. Representative images are shown. (**F**) Surface expression of CCR2 is decreased on peripheral blood neutrophils in *Tet2^–/–^* mice (*Tet2^fl/fl^* [4328 ± 449, *n* = 19]; *Tet2^–/–^* [2875 ± 407], *n* = 18), at steady-state. (**G**) Relative expression of CCL2 is lower in the lungs of *Tet2^–/–^* at steady-state (*Tet2^fl/fl^* [1.96 ± 0.35], *n* = 8; *Tet2^–/–^* [0.87 ± 0.17], *n* = 16). (**H**) Enumeration of CFUs in lungs and complete nasal turbinate (CNT) 10 days p.i. showed increased pathogen burden in the CNT of *Tet2^–/–^* mice (*Tet2^fl/fl^* [3.1 ± 0.1]; *Tet2^–/–^* [3.6 ± 0.22]). (**I**) Results from a simple linear regression between CFUs in the CNT at 10 days p.i. and whole blood inflammatory mediators showed a positive association between inflammation and pathogen burden, with a greater relationship in *Tet2^–/–^* mice. Shaded area represents 95% confidence intervals. * *P* < 0.05

To determine the clinical relevance of myeloid expansion in CHIP carriers, we challenged *Tet2^–/–^* and *Tet2^fl/fl^* mice with *Streptococcus pneumoniae*. Consistent with previous reports in WT C57Bl/6J mice, the number of *Tet2^fl/fl^* mice that became moribund and required euthanasia always did so between days 2 and 4 postinfection (p.i.), at the peak of symptom activity. Mice that survived past day 4 p.i. recovered from the infection. In contrast, morbidity and mortality were prolonged in the *Tet2^–/–^* mice, whose health continued to decline 7 days after *S*. *pneumoniae* colonization (Figure 4A), resulting in higher overall mortality (64% in *Tet2^–/–^* mice versus 18% in *Tet2^fl/fl^* mice). At 10 days p.i., the surviving *Tet2^–/–^* mice exhibited severe lung pathology including large areas of necrosis and hemorrhage, marked thickening of the bronchial mucosa, and widespread inflammatory cell infiltration into the alveoli (Figure 4, B and C), compared with the floxed control mice (*Tet2^fl/fl^*).

Investigation of differential cellular influx in the lungs of *Tet2^–/–^* mice revealed an increase in mononuclear phagocytes (F4/80^+^ macrophages, monocytes, and dendritic cells) (Figure 4D), compared with *Tet2^fl/fl^* mice. In contrast, we found that neutrophils (Ly6G^+^) were decreased in the lungs of *Tet2^–/–^* mice. Evaluation of the peripheral blood likewise showed increases in the relative number of monocytes in the *Tet2^–/–^* mice; however, surprisingly, the proportion of peripheral blood neutrophils were also higher in *Tet2^–/–^* mice (Figure 4E). We postulated that neutrophil recruitment to the lungs may be impaired in *Tet2^–/–^* mice. In order to test whether neutrophils had an intrinsic migration defect we injected CCL2 (MCP-1) into the intraperitoneal cavity. We found no differences between *Tet2^–/–^* and *Tet2^fl/fl^* mice (Supplemental Figure 1), suggesting no inherent defects in transendothelial migration. We next investigated whether lower recruitment of neutrophils in the lung might be a consequence of defective chemotactic signaling. In support of this, we found that CCL2 expression was decreased in the lungs and surface CCR2 expression was decreased on peripheral blood neutrophils in *Tet2^–/–^* mice at steady state (Figure 4, F and G). Taken together, these results indicate that *Tet2* deficiency confers an inappropriate and pathological myeloid cell response in pneumonia.

### Tet2^–/–^ mice have impaired clearance of S. pneumoniae.

To determine whether the reduced neutrophil recruitment in *Tet2^–/–^* mice altered pathogen load, we quantified CFUs from multiple organs at critical and experimental endpoint. At critical endpoint, the numbers of *S*. *pneumoniae* recovered from the complete nasal turbinates (CNT), lungs, spleens, and brain of the *Tet2^–/–^* mice were similar to the numbers recovered from the *Tet2^fl/fl^* mice (Supplemental Figure 2). In both groups, a large number of *S*. *pneumoniae* could be recovered from multiple organs, highlighting the severity of disease in these animals. In contrast, the pathogen burden at experimental endpoint (day 10 p.i.) was significantly different between *Tet2^–/–^* and *Tet2^fl/fl^* mice. The *Tet2^–/–^* mice that survived to day 10 p.i. had higher counts of *S*. *pneumoniae* in the CNT, compared with the *Tet2^fl/fl^* mice (Figure 4H). A similar trend was observed in the lungs, although this did not reach statistical significance. These data suggest that *Tet2^–/–^* mice are less successful at clearing *S*. *pneumoniae*, despite the increased number of mononuclear phagocytes.

To address if the impaired clearance of *S*. *pneumoniae* in *Tet2^–/–^* mice was accompanied by excessive or unregulated inflammation that is typical of sepsis (38), we correlated the main inflammatory mediators *TNF, IL6, IFN-*γ*, MCP1*, and *IL1-*β in circulation with corresponding CFUs in the CNT at day 10 p.i. There was a significant positive relationship between CFUs and all inflammatory mediators in the CNT (Figure 4I). Similar results were observed in the lungs (Supplemental Figure 3). These data provide evidence that an exaggerated inflammatory response accompanies reduced bacterial clearance.

### Tet2^–/–^ neutrophils have impaired motility, phagocytosis, and NET formation.

**Figure 5:**
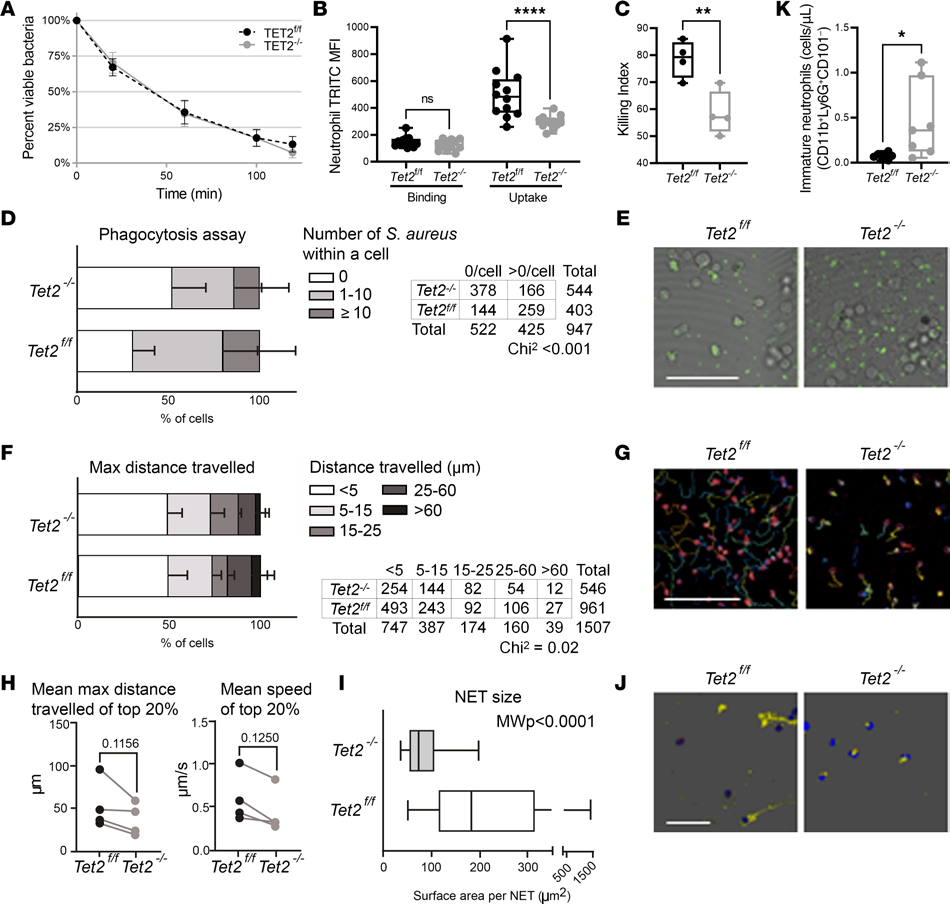
Loss of *Tet2* impairs bactericidal capacity of neutrophils. (**A**) There were no differences in bacterial killing between Tet2^fl/fl^ and Tet2^–/–^ BM-derived macrophages. (**B**) Bacterial binding and uptake, measured with pHrodo-Red-labeled *Streptococcus pneumoniae,* showed decreased pathogen uptake in *Tet2^–/–^* neutrophils (*Tet2^fl/fl^* [500 ± 14.6]; *Tet2^–/–^* [296 ± 14.1]) compared with *Tet2^fl/fl^* neutrophils. (**C**) Intracellular killing of engulfed *S*. *pneumoniae* was reduced in *Tet2^–/–^* neutrophils (*Tet2^fl/fl^* [78.5 ± 3.5]; *Tet2^–/–^* [58.3 ± 4.1]). (**D**–**J**) Functional assays comparing *Tet2^fl/fl^* versus *Tet2^–/–^* neutrophils over 30 minutes coculture with *Staphylococcus aureus*. *n* = 4 independent experiments each. (**D**) Phagocytosis of *S*. *aureus* was impaired in *Tet2^–/–^* neutrophils compared with *Tet2^fl/fl^* neutrophils. Mean ± SD of percentages of cells with certain counts of internalized bacteria. Significance tested with χ^2^ test. (**E**) Representative image showing GFP-labeled bacteria (green) at end of 30 minutes coincubation with neutrophils. Scale bar: 100 μm. (**F**–**H**) Migration qualities of *Tet2^–/–^* neutrophils in response to *S*. *aureus* were impaired compared with *Tet2^fl/fl^* neutrophils. (**F**) Mean ± SD of percentages of cells travelling certain max distances. Significance tested with χ^2^ test. (**G**) Representative tracks of cells over 30 minutes coincubation with *S*. *aureus*. Scale bar: 100 μm. (**H**) Mean maximum distances travelled (left) and mean speeds of top 20% of cells (right). Significance tested with paired *t* test and Wilcoxon, respectively. (**I**) Neutrophil extracellular traps (NETs) were less expansive in *Tet2^–/–^* (72 μm^2^ [range 54–106]) versus. *Tet2^fl/fl^* neutrophils (183 μm^2^ [range 115–312]). Boxes represent median [IQR] of individual NETs quantified, with minimum to maximum whiskers; Mann-Whitney test. (**J**) Representative image of NETs stained for dsDNA (Alexa568, yellow), nuclear DNA, (DAPI, blue), mitochondria (MitoTracker, red), *S*. *aureus* (GFP, green). Scale bar: 100 μm. (**K**) Immature neutrophil counts were higher in the circulation of *Tet2^–/–^* mice (0.45 ± 0.16; *n* = 7) versus *Tet2^fl/fl^* mice (0.07 ± 0.01; *n* = 7).

Next, we sought to determine if differences in CFUs reflect impaired uptake or removal of pathogen by *Tet2^–/–^* phagocytes. To test this, we examined the killing capacity of BM-derived macrophages (BMDM) isolated from *Tet2^–/–^* and *Tet2^fl/fl^* mice. No differences were observed in *S*. *pneumoniae* killing between the groups (Figure 5A). To determine whether the observed CFUs could instead be due to neutrophil impairments, we measured neutrophil binding and uptake. Despite similar bacterial binding capabilities, we observed reduced or delayed bacterial internalization in *Tet2^–/–^* neutrophils (Figure 5B), resulting in impaired killing of *S*. *pneumoniae* (Figure 5C). Using complimentary live imaging techniques, we found that, compared with *Tet2^fl/fl^, Tet2^–/–^* neutrophils phagocytosed fewer bacteria (*S*. *aureus*) (Figure 5, D and E) and moved more slowly, covering less distance (Figure 5, F–H). We also found that *Tet2^–/–^* neutrophil extracellular trap (NET) formation in response to *S*. *aureus*, was impaired (Figure 5, I and J). The *Tet2^fl/fl^* neutrophils formed large NETs whereas the *Tet2^–/–^* neutrophils were smaller in surface area with fewer spindle-like projections. Based on prior findings (39), we postulated that differences in neutrophil maturity might be responsible for reducing neutrophil phagocytic capacity and antimicrobial function. In keeping with this hypothesis, we found that *Tet2^–/–^* mice had higher numbers of immature neutrophils (CD11b^+^Ly6G^+^CD101^–^) in circulation (Figure 5K). Overall, *Tet2-*deficient murine neutrophils have compromised immune functions.

### Impaired motility and migratory gene pathways in Tet2^–/–^ neutrophils.

**Figure 6:**
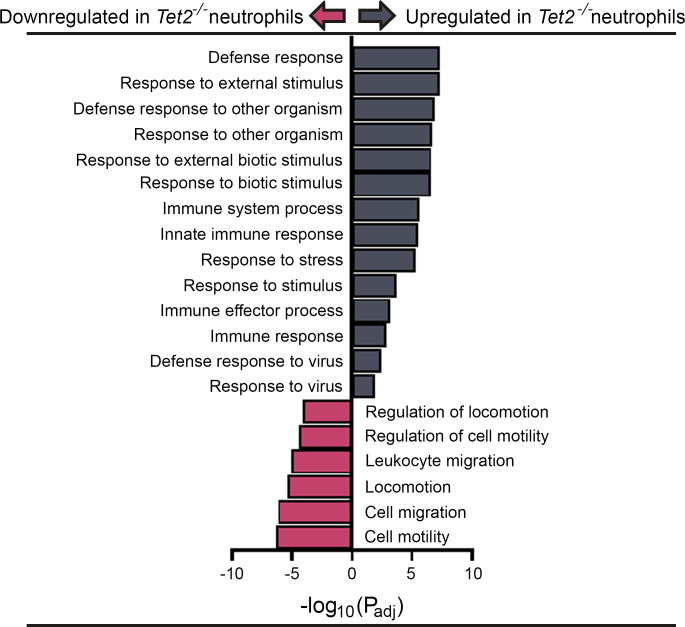
RNA-Seq of neutrophils isolated from *Tet2^–/–^* mice. Gene pathways related to immunity and defense were upregulated in neutrophils isolated from *Tet2^–/–^* mice, whereas pathways related to motility and locomotion were downregulated.

To further explore the mechanisms of *Tet2^–/–^* neutrophil dysfunction, we examined changes in gene expression using whole transcriptome sequencing (RNA-Seq). In total, we identified 130 genes that were differentially abundant in the *Tet2^–/–^* versus *Tet2^fl/fl^* control neutrophils. Specific pathways highlighted in *Tet2^–/–^* neutrophils demonstrate a clear proinflammatory signature, with enrichment of 40 genes involved in immunity and defense pathways (Figure 6). Strikingly, the remaining 99 genes were significantly downregulated *Tet2^–/–^* neutrophils. These *Tet2^–/–^*-specific genes were determined to be central in motility and migratory pathways. Taken together, these molecular data provide evidence that *Tet2^–/–^* neutrophils mediate a paradoxical state of enhanced inflammation but also reduced phagocytic capacity, which has clinical consequences during infection.

## Discussion

There is emerging evidence that individuals with clonal hematopoiesis are at increased risk of severe infections (20), but the mechanisms and pathways of susceptibility are not known. Consistent with previous reports (17), we show that murine hematopoietic *Tet2-*KO and *TET2*-mutant CHIP promotes changes in the hematopoietic system in both mice and humans, resulting in dysregulated myelopoiesis. In mice, the proportion of BM HSCs committed to the myeloid lineage were consistently higher in *Tet2^–/–^* animals compared with control animals. This was accompanied by an increase in CCR2 expression, which promotes the release of monocytes into circulation (36). We showed that in both mice and humans, inflammatory subsets of monocytes (Ly6C^hi^ and classical monocytes, respectively) increased with *Tet2/TET2*-inactivation. In addition, we demonstrate that like *Dnmt3a-*mutant cells (40), myeloid cells from *Tet2^–/–^* mice, have a greater proclivity to become hyper-activated in response to inflammatory insults, such as TNF, resulting in increased production of inflammatory cytokines and myeloid expansion. We postulate that the increased response could be mediated through increased TNFR2 expression, (40) although this remains to be formally demonstrated. Taken together, these data support the notion that defects in *Tet2* promote a hyper-inflammatory immunogenic profile that may contribute to worse outcomes of infections (20) and other CHIP comorbid conditions (41).

Clearance of a pneumococcal pneumonia requires an appropriate innate immune response, particularly by phagocytic cells such as neutrophils and monocytes. Despite the importance of these myeloid lineage cells in controlling pathogen burden, an exacerbated inflammatory response can cause lung damage and pathology and may become the primary source of morbidity and mortality (42). Impairment of innate resolution in the lungs has been linked to poorer outcomes in several models of bacterial and viral pulmonary infections (43–45). Thus, successful control of pneumococcal infections requires not only neutrophil and monocyte clearance of the invading pathogens, but also resolution of these immune cells to prevent tissue damage. Here, we show that *Tet2^–/–^* mice have a pronounced increase in pulmonary monocytes following infection, which failed to resolve by day 10 p.i. We show that the persistence of these highly inflammatory cells resulted in lung damage without effective pathogen clearance.

The inability of *Tet2^–/–^* mice to effectively clear *S*. *pneumoniae* was attributed to impaired neutrophil function. Our results show that CC chemokine receptors and their ligands, which are necessary for neutrophil mobilization and recruitment to the lungs during infection (46–48), are down regulated in human CHIP carriers (e.g., CXCL1, CXCL5) and mice (e.g., CCR2, lung CCL2). As we did not see any differences in neutrophil transendothelial migration toward an administered chemoattractant, we attribute the reduced numbers of neutrophils in the lungs to reduced chemotactic signaling, rather than inherent defects in recruitment. It is also possible that the neutrophils were more efficiently cleared from the lungs by the increased presence of monocytes, but additional studies are needed to fully address this question. Nevertheless, in humans with CHIP we also found decreased expression of CD64, an Fc receptor associated with neutrophil activation and phagocytosis. Previous studies have demonstrated that a worse prognosis and survival in patients correlated with decreased expression of CD64 and with impaired neutrophil phagocytic activity (49). The authors proposed that neutrophil phagocytic activity may serve as a prognostic indicator of sepsis. Excessive immature forms of neutrophils are also related to clinical deterioration in patients with sepsis (39). Consistent with these findings, we show that *Tet2^–/–^* mice had an increase in immature neutrophils in peripheral blood and that the neutrophils isolated from *Tet2^–/–^* mice had reduced or delayed bacterial phagocytic activity and cell motility. We also show that the expulsion of neutrophil nuclear contents to form NETs was impaired in *Tet2^–/–^* neutrophils ex vivo, which may be due to reduced CXCL1-CXCR2 signaling (47). Further characterization of *Tet2^–/–^* neutrophils using RNA-Seq revealed that gene pathways important for phagocytic functions (i.e., motility) were reduced, rendering *Tet2^–/–^* mice highly susceptible to *S*. *pneumoniae*. Overall, we demonstrate that *Tet2* mutations drive known sepsis features, such as cytokine-driven hyperinflammatory response and immunosuppression, characterized by reduced activity of phagocytes (50). These findings broaden our understanding of the role of Tet enzymes in regulating infectious disease outcomes and highlight innate immune dysfunction as a key contributor to pneumonia risk in mutant-*TET2* CHIP carriers.

## Methods

### Sex as a biological variable.

Our study examined male and female animals, and similar findings are reported for both sexes.

### UK Biobank cohort.

The UK Biobank is a large observational cohort of individuals residing in the United Kingdom; it contains baseline demographic information such as age, sex, and health habits as well as ongoing clinical health records. From this data set, we determined the CHIP status for all participants with available whole exome data (*n* = 453,510), as detailed previously (51). Individuals with a history of hematologic cancer at enrollment are excluded from the CHIP data set. Incident pneumonia was defined using the UKB field IDs 131,446 through 131,457, which correspond to the dates of first report of pneumonia (ICD-10 codes J13–J18) from any source (death register, primary care, hospital admissions, or self report).

### Human participants and CHIP genotyping.

Community-dwelling research participants were recruited from the Greater Hamilton Area between November 2017 and January 2020. Venous blood was drawn in anticoagulant-free vacutainers for the isolation of serum and in heparin-coated vacutainers for the experiments that required viable leukocytes (52). Participant demographic information (age, sex, height) and health status (components of the Charlson Comorbidity Index [CCI], BMI, medication history, vaccination history, and frailty scores) were provided at the time of sample collection. Only participants who had not required antibiotics within 2 weeks of sample collection were included in this analysis. CHIP status was determined by applying a successful 48-gene, targeted, ion-torrent–based sequencing approach to isolated genomic DNA from peripheral blood mononuclear cells with a VAF of at least 2%, as previously described (33, 51).

### Animals.

*Tet2^fl/fl^* (B6;129S-Tet2tm1.1Iaai/J) and *Vav1-iCre* (B6.Cg-Tg(Vav1-icre)A2Kio/J) mice were bred at Queen’s University to produce *Tet2^fl/fl^ Vav1-icre^–/–^* (*Tet2^fl/fl^*) and *Tet2^fl/fl^ Vav1-icre^+/–^* (*Tet2^–/–^*) genotypes (exon 3 of *Tet2* gene is floxed) in the hematopoietic system. For infection experiments, mice were transported to the McMaster Central Animal Facility and maintained under a 12-h light-dark cycle at 22 ± 2°C and 55 ± 5% air humidity. Mice had ad libitum access to Teklad irradiated global 14% protein diet (Envigo) and autoclaved reverse osmosis water. Any mice that developed tumors during the entire period of observation were omitted from analyses.

### Streptococcus pneumoniae infection.

8–10-month-old male and female mice were intranasally inoculated with 40 μL 10^4^ CFU of *S*. *pneumoniae* P1542, serotype 4, as described previously (53). While risk of pneumococcal disease increases with age, we were unable to use aged mice (over 18 months) due to the increased incidence of tumor formation in *Tet2^–/–^* mice. Mice were given ad libitum access to Ensure and HydroGel and the cages were placed on heating pads for the 10-day duration of the infection (experimental endpoint at 10 days p.i.). Mice were monitored 3 times daily, and if any mouse became moribund (critical endpoint) or lost 20% body weight, it was immediately sacrificed.

### Sample collection and tissue processing.

Prior to mouse euthanasia, retro-orbital blood collections were used for immunophenotyping, whole blood stimulations, LeukoSpins, and sera collection. Mice were then exsanguinated, and laparotomies were performed. Tissues were immediately collected and fixed in 10% neutral buffered formalin (48 hours, 4°C [Thermo Fisher Scientific]) for H&E staining and IHC. Murine BM progenitors were collected from the vertebral column for immunophenotyping or differentiated into macrophages for functional assays, as have been previously published (54).

### Histopathological scoring and IHC.

The lung histopathology was conducted by 2 blind observers as previously described (55). The Core Histology Facility at the McMaster Immunology Research Centre performed the lung IHC. Briefly, paraffin-embedded tissue sections were dewaxed, hydrated, and treated with Bond ER2 (Leica) for epitope retrieval. Slides were then stained with anti-F4/80 (Bio-Rad, MCA-497R; 1:1,000 dilution) or anti-Ly6G (BioLegend, 127602; 1:1,000 dilution) antibodies using the Leica Bond Rx Automated Stainer and the Bond Polymer Refine detection kit. An external rabbit anti-rat secondary antibody (Vector labs, BA-4001), which was preabsorbed against mouse, was then used to visualize the mononuclear phagocytes (F4/80^+^ macrophages, monocytes, and dendritic cells) and neutrophils (Ly6G^+^).

### Immunophenotyping by flow cytometry.

For whole blood and BM immunophenotyping, 100 μL of heparinized blood and isolated BM were incubated with antibodies (Supplemental Table 2) for 30 minutes at room temperature and then incubated in a 1× dilution of 1-step Fix/Lyse buffer (eBioscience) for 10 minutes. Cells were washed and resuspended in fluorescence-activated cell sorting (FACS) wash buffer (PBS, 0.5% BSA, 2 mM EDTA) prior to analysis. For intracellular markers, samples were incubated in the presence of 50 ng/ mL LPS or vehicle control for 4 hours. Following incubation, cells were initially surface stained with antibodies and then intracellular staining was performed after 30 minutes permeabilization at room temperature with 1× Intracellular Staining Permeabilization Wash Buffer (BioLegend), as previously described (24). Absolute cell counts were determined using CountBright Absolute Counting Beads (Life Technologies).

Gating strategies for HSC, monocyte, and neutrophil populations are provided in Supplemental Figures 4–6, for humans and mice. All fluorescence gates were set using appropriate isotype controls or FMOs and compensation of spectral overlap was performed for all fluorochromes. Flow cytometry was performed on a Cytoflex and analyzed using FlowJo software (version 10.7.1, Becton Dickinson & Company). Data is reported as percent positive or absolute count for each cell subset.

### Measurement of cytokine production.

Serum IFN-γ, IL10, IL1-β, IL6, MCP1, and TNF were measuring using high-sensitivity ELISA per the manufacturer’s recommendations (Meso Scale Discovery Cat. No. K15069L-1). Lung TNF, IL6, CCL2, and CXCL1 were measured by quantitative PCR (qPCR) as previously reported.

### Whole blood bacterial binding and uptake assay.

The bacterial binding and uptake assay using whole blood has been described previously (56). Briefly, to visualize *S*. *pneumoniae* binding and engulfment capacity of phagocytes, TRITC-labeled bacteria were incubated with whole blood for 1 hour at an MOI of 50. Leukocytes were fixed, stained, and analyzed by flow cytometry.

### BM-derived macrophage killing assay.

For the macrophage killing assay, 5 × 10^5^ BM-derived macrophages were preincubated with *S*. *pneumoniae* at a multiplicity of infection (MOI) of 100 bacteria per macrophage for 60 minutes at 37°C with gentle shaking to allow for phagocytosis. Viable CFUs of surviving bacteria were determine by culturing supernatants on TS agar plates.

### Neutrophil phagocytosis and migration assay.

BM was isolated from age-sex matched 2-to-4-month-old mice as previously described (57), and neutrophil enrichment followed subsequently using the EasySep Mouse Neutrophil Enrichment Kit (StemCell Technologies) according to the manufacturer’s instructions. The resulting neutrophil sample purity was assessed by a hematopathologist using H&E staining, and CD11b and Ly6G flow cytometric analysis, confirming expected purity. Neutrophils were stained with Mitotracker Deep Red (Thermo Fisher Scientific) and Nuc Blue (Thermo Fisher Scientific). 5 × 10^5^ cells in 0.5 mL of phenol red-free RPMI (Gibco) supplemented with 10% pooled mouse serum were plated per 35 mm glass-bottom imaging dish (Mattek corporation). GFP-labeled *Staphylococcus aureus* was grown overnight to an OD_600_ of 0.5 and subsequently cocultured with neutrophils at a ratio of 10:1. Time-9lapse imaging was performed using the SP8-X confocal scanning microscope (Leica) and 4 fields of view were captured over 30 minutes per sample. Phagocytosis, migration, and NET surface area were analyzed in FIJI after stitching the 4 fields of view together. *Staphylococcus aureus* counts per cell were performed. Migration analysis was performed using the TrackMate plugin, and max distance (between farthest 2 points) of each cell track and average speed along each track were quantified. To determine differences in transendothelial migration of circulating leukocytes, we intraperitoneally injected mice with 100 nM MCP-1/CCL2 and measured leukocyte recruitment after 4 hours, as previously reported (24).

### Sequencing of RNA.

RNA was extracted from neutrophils, as previously described (58). Briefly, RNA was extracted using the RNeasy Mini Kit (Qiagen) following the manufacturer’s instructions. Aliquots of RNA were quantified on the Bioanalyzer RNA 6000 Nano kit (Agilent) and stored at –80°C for quantitative reverse-transcription PCR (qRT-PCR) analysis. 120–300 ng of RNA was used to generate libraries for 2 independent RNA-Seq experiments by the QuantSeq 3′ mRNA-Seq library prep kit for Illumina (Lexogen). Libraries were generated by 3′ poly A tail capture for quantification of mRNA transcripts. Sequencing was performed on the Illumina NextSeq platform at the QCPU core facility (Single end, 75 bp read length, 130 million reads).

### Bioinformatic analysis of RNA-Seq data.

The raw Fastq files were demultiplexed and trimmed using Fastp (59). This was followed by alignment of the reads, which was performed on the Queen’s Center for Advanced Computing computer cluster. The quality of reads was analyzed using the FastQC module. Alignment of reads was performed by the STAR (Spliced Transcripts Alignment to a Reference) (60) tool and the trimmed fastq files were aligned to the current mouse gene assembly, GRCm38, using the GENCODE VM23 annotation. Normalization of sequence depth, distribution, correlation, batch effects, and outliers were then all performed using the DESeq2 analysis program (61). Tables of gene counts were then created. Finally, analysis of gene expression was performed in R using DESeq2 (61). The analysis focused on the differences between *Tet2^fl/fl^* versus *Tet2^–/–^* neutrophils under baseline (no treatment) conditions. This is because we were interested in seeing the changes in neutrophil gene expression and function in the steady state. The lists of significantly upregulated and downregulated genes were then merged to conduct pathway enrichment analysis/genome ontology (GO), which was performed using the g:Profiler (62). Differential expression counts were then visualized through Volcano Plots (Supplemental Figure 7) and normalized counts were graphed using Prism GraphPad. Students *t*-tests were used to calculate significance for normalized counts.

### Statistics.

Statistical analyses were performed in GraphPad Prism V9.2 or R 4.1.2. For UK Biobank data analysis. Cox Proportional Hazards regression were used to estimate the risk of incident infections by CHIP and *TET2* status. Covariables of age, age^2^, sex, smoking history, and 10 principal components of genetic ancestry were added to the model. In a separate model, we added an interaction term from a common SNP in the IL6 receptor that is associated with lower IL6 signaling (rs2228415). For the animal models, the data were tested for normality using Shapiro-Wilk test, and a 1-way ANOVA was used to compare mean differences among groups with Benjamini-Hochberg multiple testing correction applied as needed. For comparisons between 2 groups, significance was calculated using a 1-tailed Student’s *t* test or Mann-Whitney test where appropriate. The results are expressed as the mean value with SEM, unless otherwise stated. Genes and GO terms are reported for adjusted *P* values and FDR values < 0.05. A *P* value < 0.05 was considered significant.

### Data availability.

Data sets supporting the results of this article are available in the Open Science Framework (OSF) database repository at https://osf.io/bcta2/

### Study approval.

The animal ethics have been approved by the McMaster Animal Research Ethics Board (no. 17-05-19) and Queen’s University Animal Care committee (Protocol 2021-2128) and performed in accordance with the Canadian Council on Animal Care guidelines. All human protocols were approved by the Hamilton Research Ethics Board (#4640) and Queen’s University Health Sciences and Affiliated Teaching Hospitals (HSREB) (PATH-181-18). Informed consent was received prior to participation.

## Author contributions

DMEB and MJR conceived and funded the experiments. DMEB, MJR, and CQ designed the experiments. CQ, END, AEK, and JAB performed flow cytometry. KZ performed qPCR and assisted with functional assays. CQ and END performed animal experiments and tissue collection. CV, MB and AGB performed UK Biobank data analyses. EKC, YZL, SS, AJMM, JM, KJDS, CH, and SLA performed and supported murine neutrophil functional and transcriptomic studies at Queen’s. CQ performed IF, histological evaluation, and critically evaluated all the data. CQ and AEK performed LeukoSpin analysis. CQ, MJR, and DMEB wrote the paper. All coauthors edited the paper.

## Supplementary Material

Supplemental data

Supporting data values

## Version 1 04/04/2024

In-Press Preview

## Version 2 06/03/2024

Electronic publication
