# Supplementary material for: Neutrophil-mediated innate immune resistance to bacterial pneumonia is dependent on Tet2 function
Source: J Clin Invest. 2024 Apr 4;134(11):e171002. doi: 10.1172/JCI171002 (PMC11142737; doi:10.1172/JCI171002)
Supplement: Supplemental data [file jci-134-171002-s021.pdf]

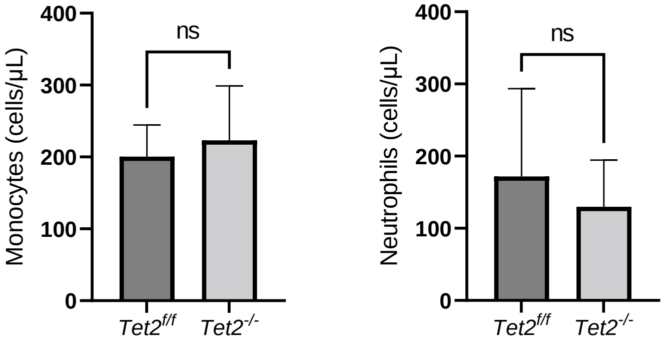

Figure S1. There were no differences in the number of cells recruited to the peritoneum 4 hours after administration of 100 nM CCL2 in *Tet2*$^{-/-}$ (n=8) and *Tet2*$^{f/f}$ (n=8) mice. Mean ± SEM.

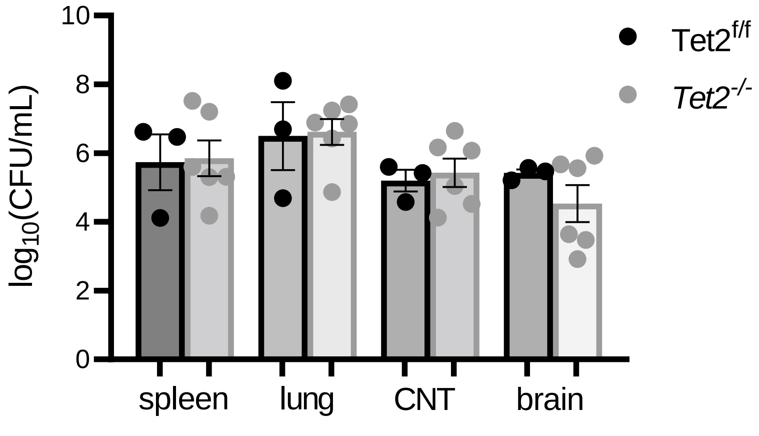

Figure S2. Enumeration of *Streptococcus pneumoniae* CFU in spleen, lung, complete nasal turbinate (CNT) and brain at critical endpoint in mice.

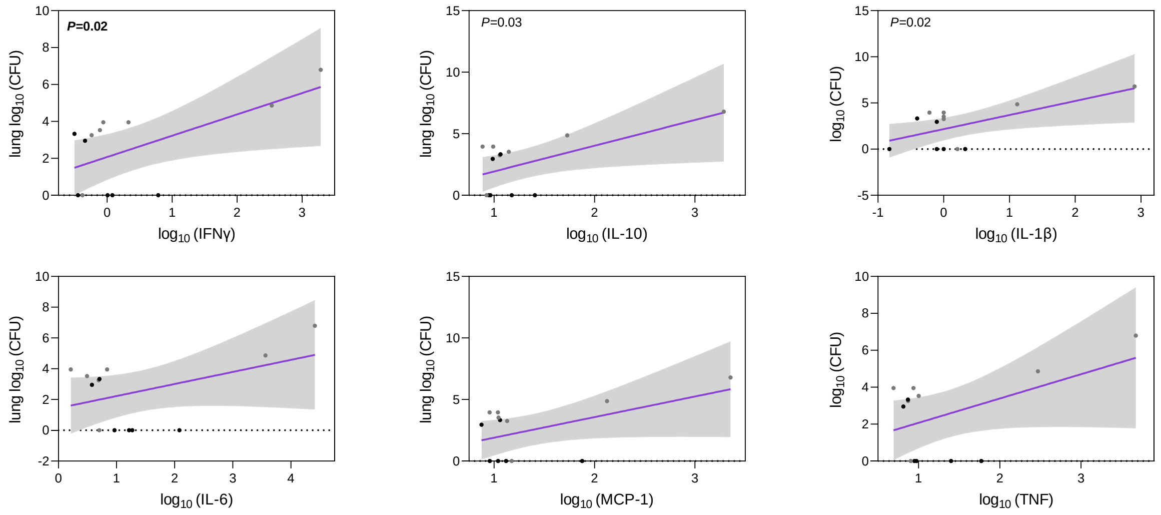

Figure S3. Results from simple linear regression between CFUs in the lung at 10 days p.i. and whole blood inflammatory mediators (IFNγ, IL10, IL1β, IL6, MCP1, and TNF) showed positive associations between inflammation and pathogen burden.

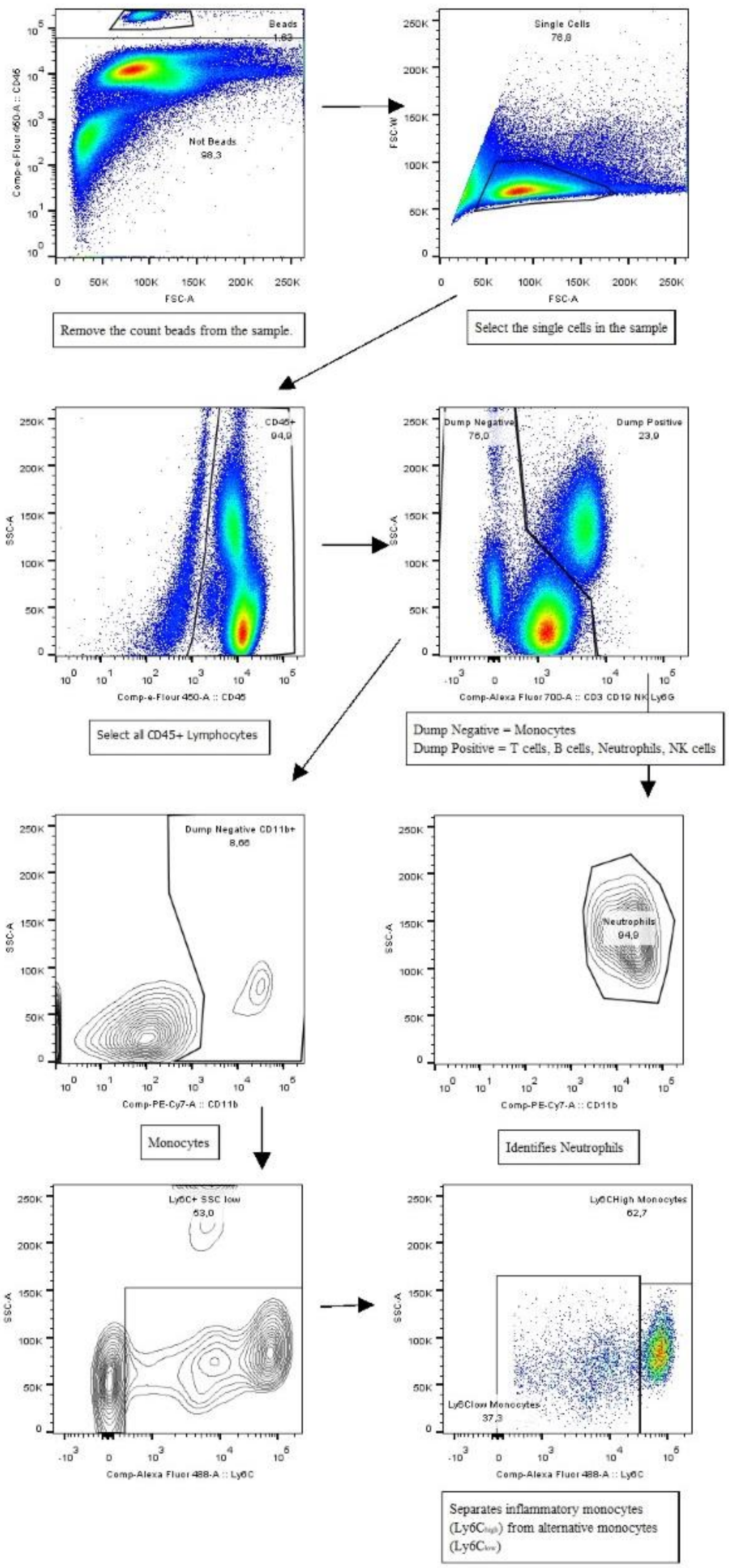

Figure S4. Gating strategy for myeloid cell populations in mice.

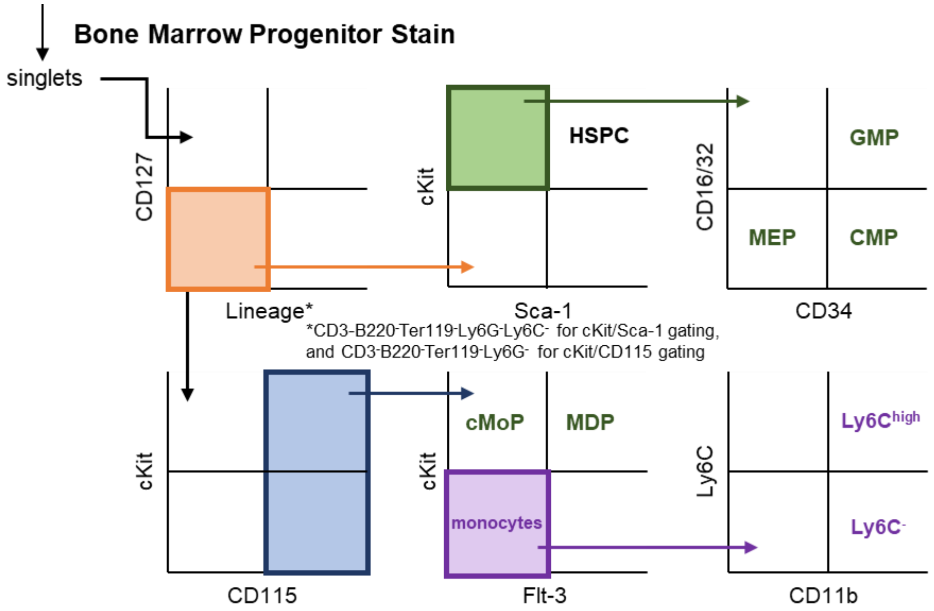

Figure S5. Gating strategy for bone marrow progenitor cells.

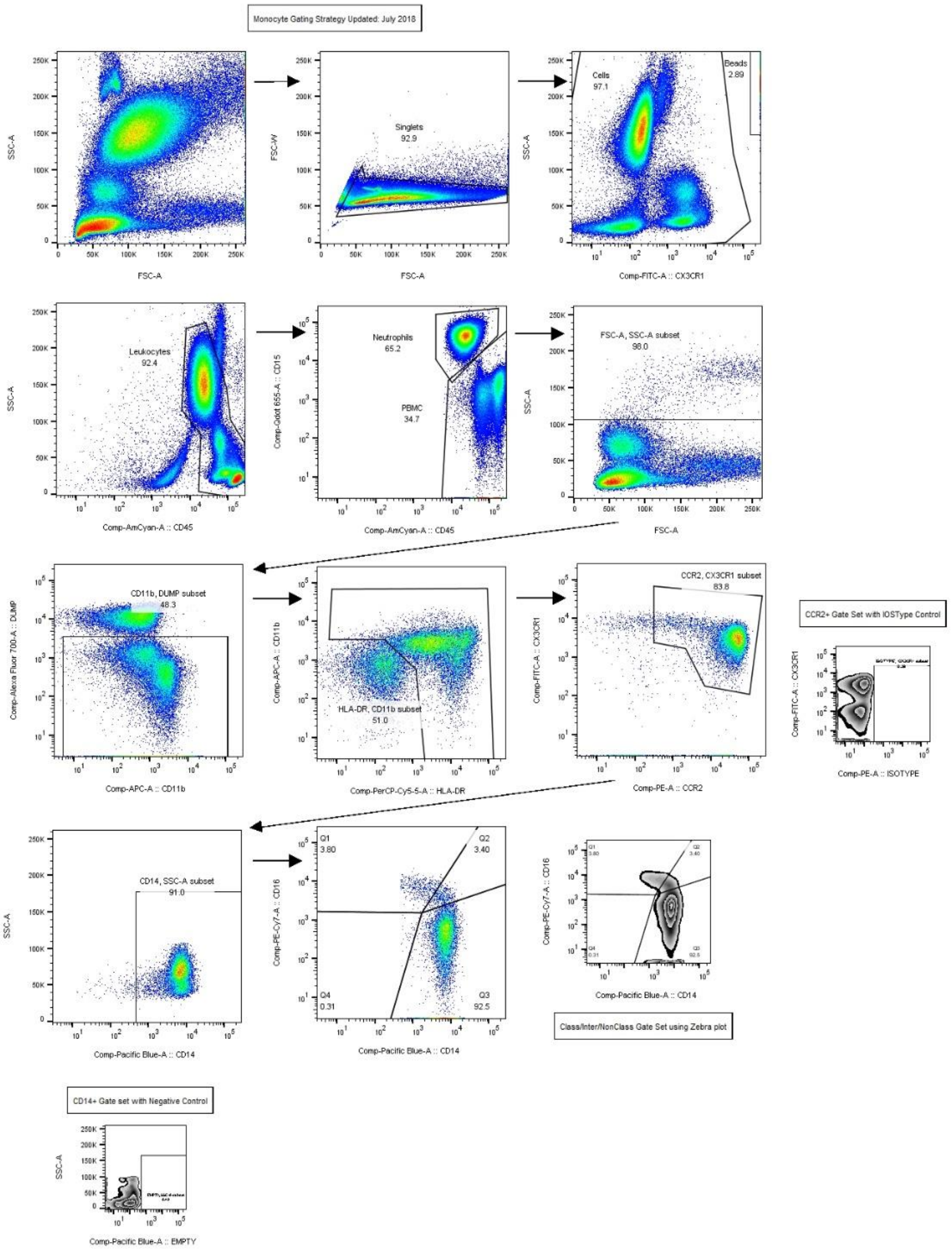

Figure S6. Gating strategy for human monocyte subsets.

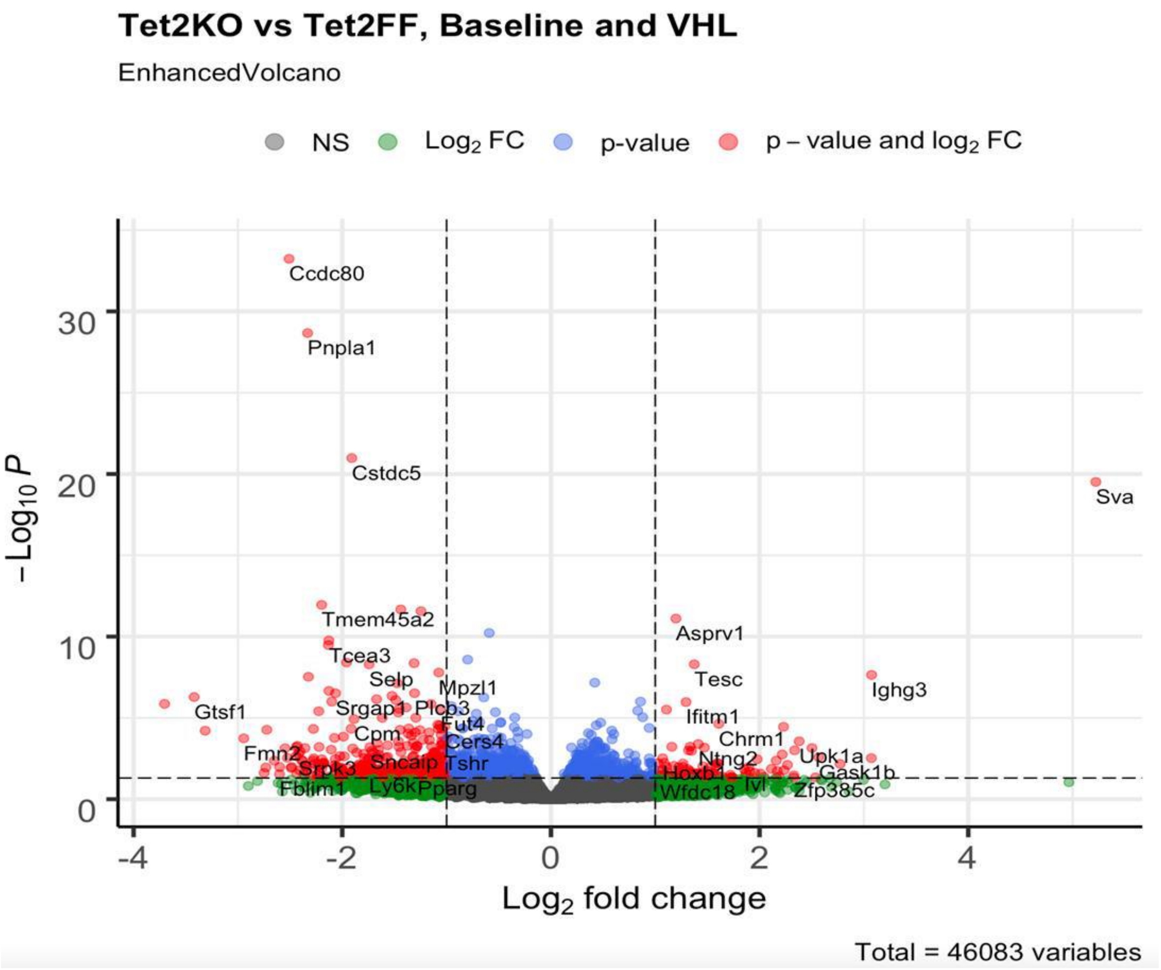

Figure S7. Volcano plot depicting differential expression of genes in *Tet2*$^{-/-}$ vs. *Tet2*$^{f/f}$ neutrophils. The LogFC cuttoff is set at Log_2=1, and the *P* value cutoff is set at *P*= 0.05. Genes on the right side of the plot are upregulated in the *Tet2*$^{-/-}$ neutrophils, while the genes on the left of the plot are downregulated in the *Tet2*$^{-/-}$ cells. Differential expression analysis was done using DESeq2 in R. Normalization was done internally through DESeq2.

Table S1. Summary of group characteristics

	No CHIP (n=16)	CHIP (n=6)
Age (yrs)	80.6 (12.1)	83.5 (12.5)
BMI (kg/m2)	26.8 (2.7)	25.4 (1.9)
Sex (n=F:M)	12:4	2:4
	% Positive (n)	
CMV positive	87.5 (14)	66.6 (4)
Smoker	6.3 (1)	16.6 (1)
**Comorbidities**		
Hypertension	18.7 (3)	16.6 (1)
Diabetes mellitus	6.2 (1)	0 (0)
Cardiovascular	0 (0)	16.6 (1)
Hyperthyroidism	6.3 (1)	0 (0)
Mood	0 (0)	16.6 (1)
Joint	18.7 (3)	33.3 (2)
GI	18.7 (3)	16.6 (1)
Respiratory	31.2 (5)	16.6 (1)
Osteoporosis	6.2 (1)	0 (0)
Other	6.2 (1)	0 (0)
Cancer history	18.7 (3)	33.3 (2)

Data showing mean (SD) unless otherwise stated. Abbreviations: *BMI* body mass index, *GI* gastrointestinal, *CMV* cytomegalovirus. Cardiovascular conditions included: hyperlipidemia. Respiratory conditions included: asthma, interstitial lung disease. GI conditions included: gastrointestinal reflux disease. Joint comorbidities included: rheumatoid arthritis and osteoarthritis. Other comorbidities included: Parkinson’s disease. Comparisons of comorbidities were performed using a Fisher’s exact test.

Table S2: Fluorophore-conjugated monoclonal antibodies used in flow cytometry

Cell surface marker	Fluorophore	Clone	Company	Cat No.
Mouse myeloid staining				
CD45	eF450	30-F11	invitrogen	48-0451-82
CD11b	PECy7	M1/70	invitrogen	25-0112-82
Ly6C	AF488	HK1.4	BioLegend	128022
CCR2	PE	475301	R&D systems	FAB5538P
F4/80	APC	BM8	eBioscience	17-4801-82
CD3	AF700	17A2	invitrogen	56-0032-82
CD19	AF700	eBio1D3	invitrogen	56-0193-82
NK	AF700	PK136	invitrogen	56-5941-82
Ly6G	AF700	1A8	BioLegend	127622
CX3CR1	BV650	SA011F11	BioLegend	149033
Neutrophil Maturation				
CD45	eF450	30-F11	invitrogen	48-0451-82
CD11b	APCCy7	M1/70	eBioscience	25-0112-82
Ly6C	AF488	HK1.4	BioLegend	128022
CD101	PE	Moushi101	eBioscience	12-1011-80
CCR2	BV785	SA203G11	BioLegend	150621
CX3CR1	BV650	SA011F11	BioLegend	149033
CD3	AF700	17A2	invitrogen	56-0032-82
CD19	AF700	eBio1D3	invitrogen	56-0193-82
NK	AF700	PK136	invitrogen	56-5941-82
Ly6G	PECy7	1A8	BioLegend	127617
Mouse Progenitor staining				
CD115 (M-CSF R)	AF488	AFS98	Invitrogen	53-1152-82
CD135 (Flt-3/Flk-2)	PE	A2F10	BioLegend	135305
Sca-1 (Ly6A)	PE-Dazzle 594	D7	BioLegend	108137
CD127 (IL7R)	PerCP-Cy5.5	SB/199	BioLegend	121114
Ter119	PE-Cy7	Ter119	eBioScience	25-5921-81
CD3	PE-Cy7	145-2C11	Invitrogen	25-0031-82
B220	PE-Cy7	RA3-62B	BioLegend	103222
CD117 (cKit)	BV421	2E8	BioLegend	105827
Ly6C	BV510	HK1.4	BioLegend	128033
CD16/32	BV711	93	BioLegend	101377
CD34	AF 647	HM34	BioLegend	128606
Ly6G	AF 700	1A8	BioLegend	127622
CD11b	APC-Cy7	M1/70	BD	557657
Human myeloid staining				
CD45	BV510	HI30	BioLegend	304036
CD16	PE-Cy7	CB16	eBioscience	25-0168-42
CD14	BV421	M5E2	BioLegend	301830
CCR2	PE	K036C2	BioLegend	357205
CD11b	APC	ICRF44	BD Biosciences	561015
HLA-DR	PerCPCy5.5	LN3	eBioscience	45-9956-42
CX3CR1	FITC	2A9-1	Cedarlane	D070-4
CD15	BV650	SSEA-1	BioLegend	323033
CD3	AF700	UCHT1	BD Biosciences	557943
CD56	AF700	5.1H11	BioLegend	362522
CD19	AF700	HIB19	eBioscience	56-0199-42

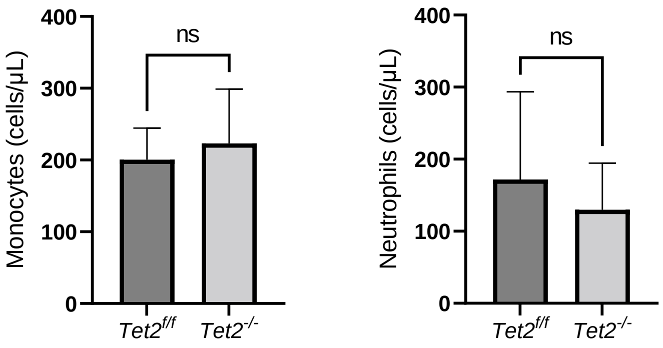

Figure S1. There were no differences in the number of cells recruited to the peritoneum 4 hours after administration of 100 nM CCL2 in *Tet2*$^{-/-}$ (n=8) and *Tet2*$^{f/f}$ (n=8) mice. Mean ± SEM.

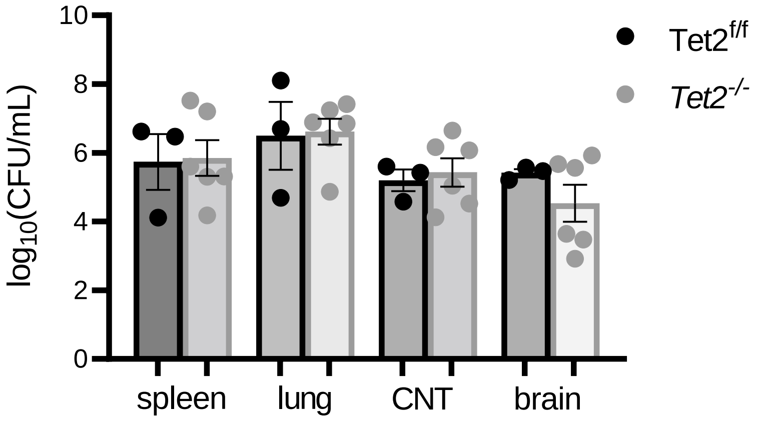

Figure S2. Enumeration of *Streptococcus pneumoniae* CFUs in spleen, lung, complete nasal turbinate (CNT) and brain at critical endpoint in mice.

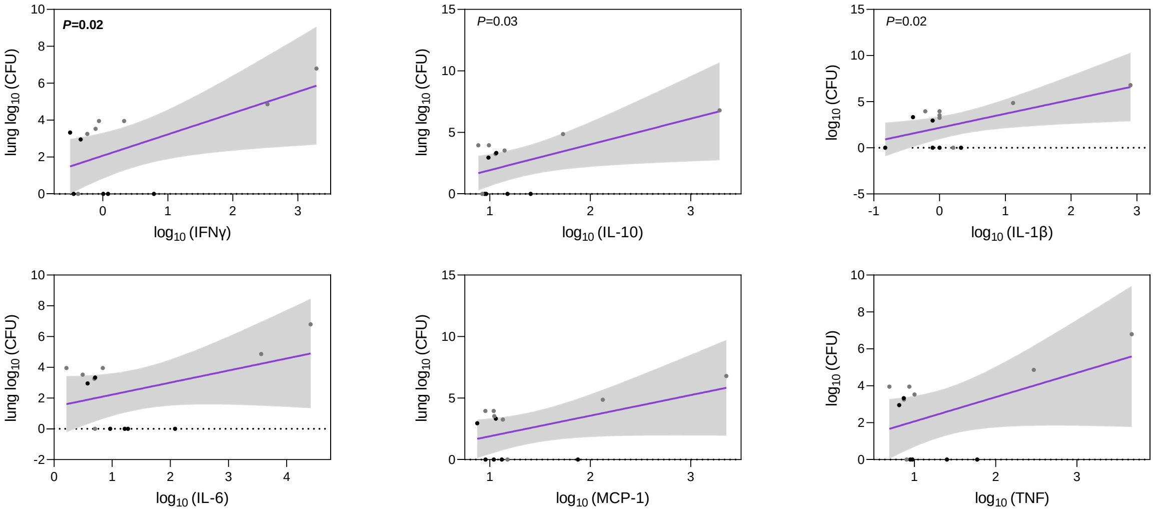

Figure S3. Results from simple linear regression between CFUs in the lung at 10 days p.i. and whole blood inflammatory mediators (IFNγ, IL10, IL1β, IL6, MCP1, and TNF) showed positive associations between inflammation and pathogen burden.

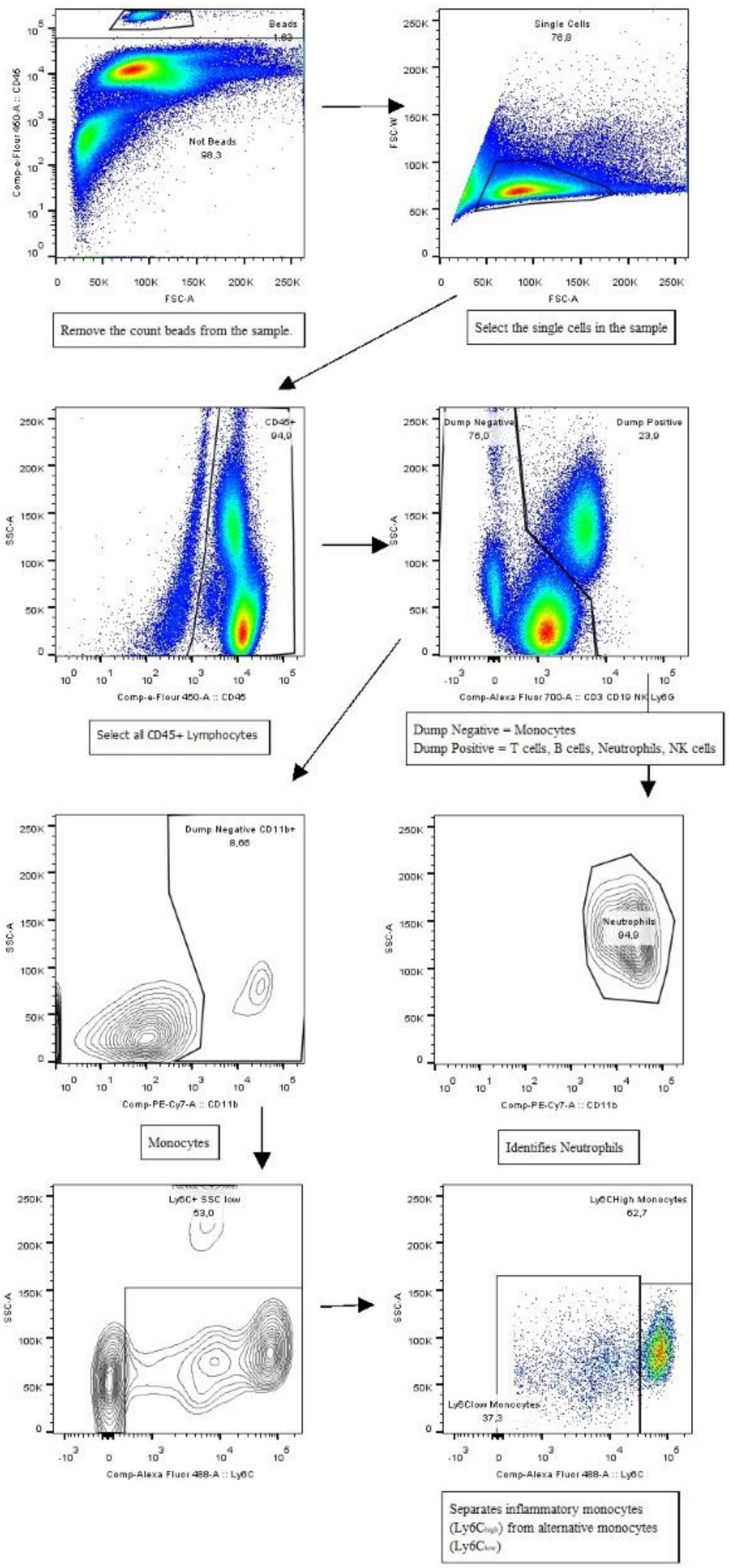

Figure S4. Gating strategy for myeloid cell populations in mice.

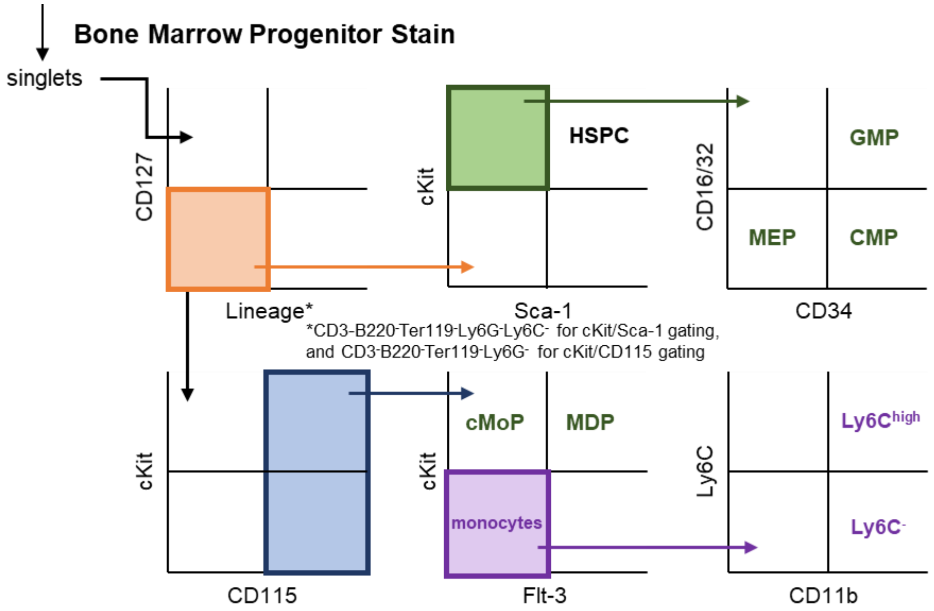

Figure S5. Gating strategy for bone marrow progenitor cells.

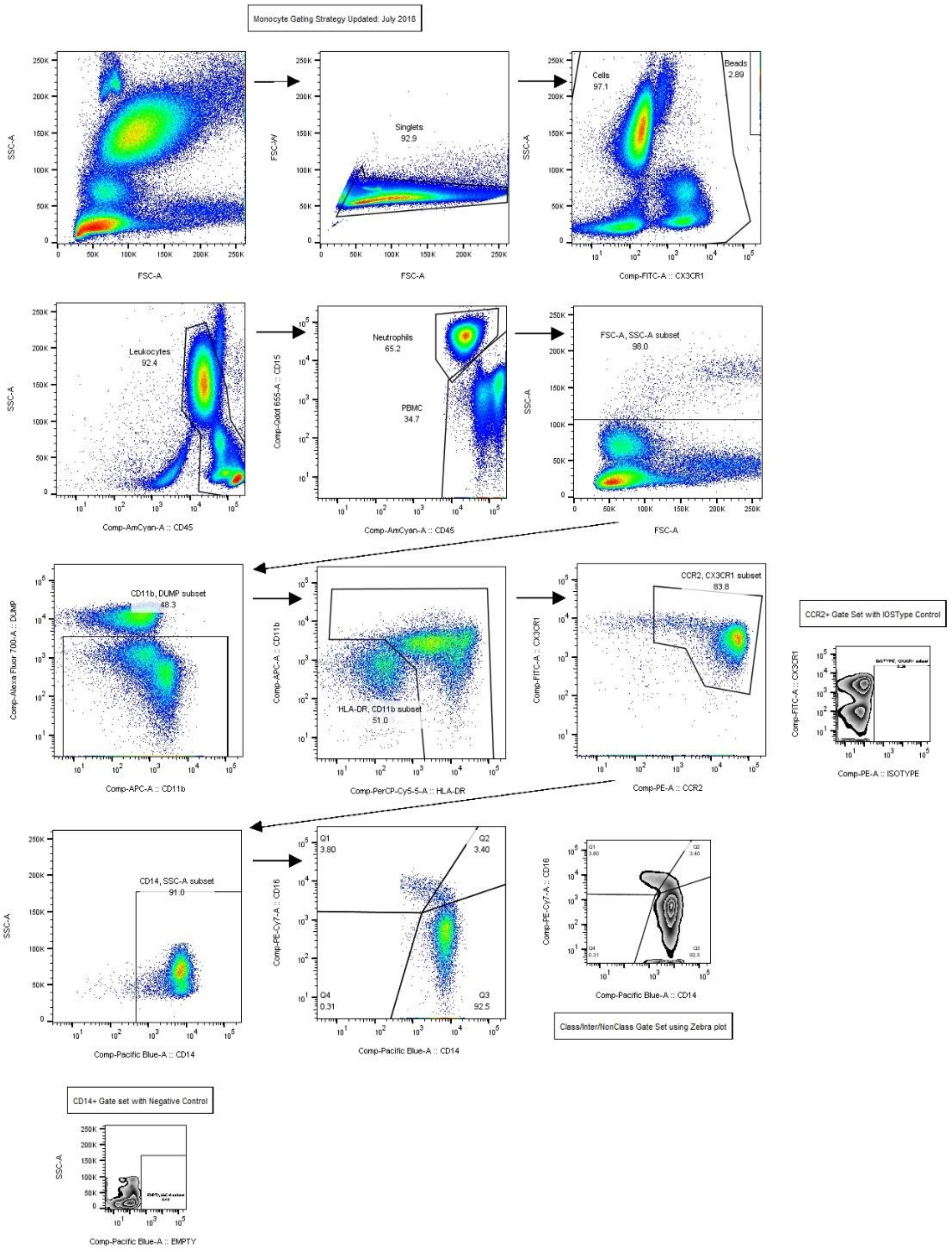

Figure S6. Gating strategy for human monocyte subsets.

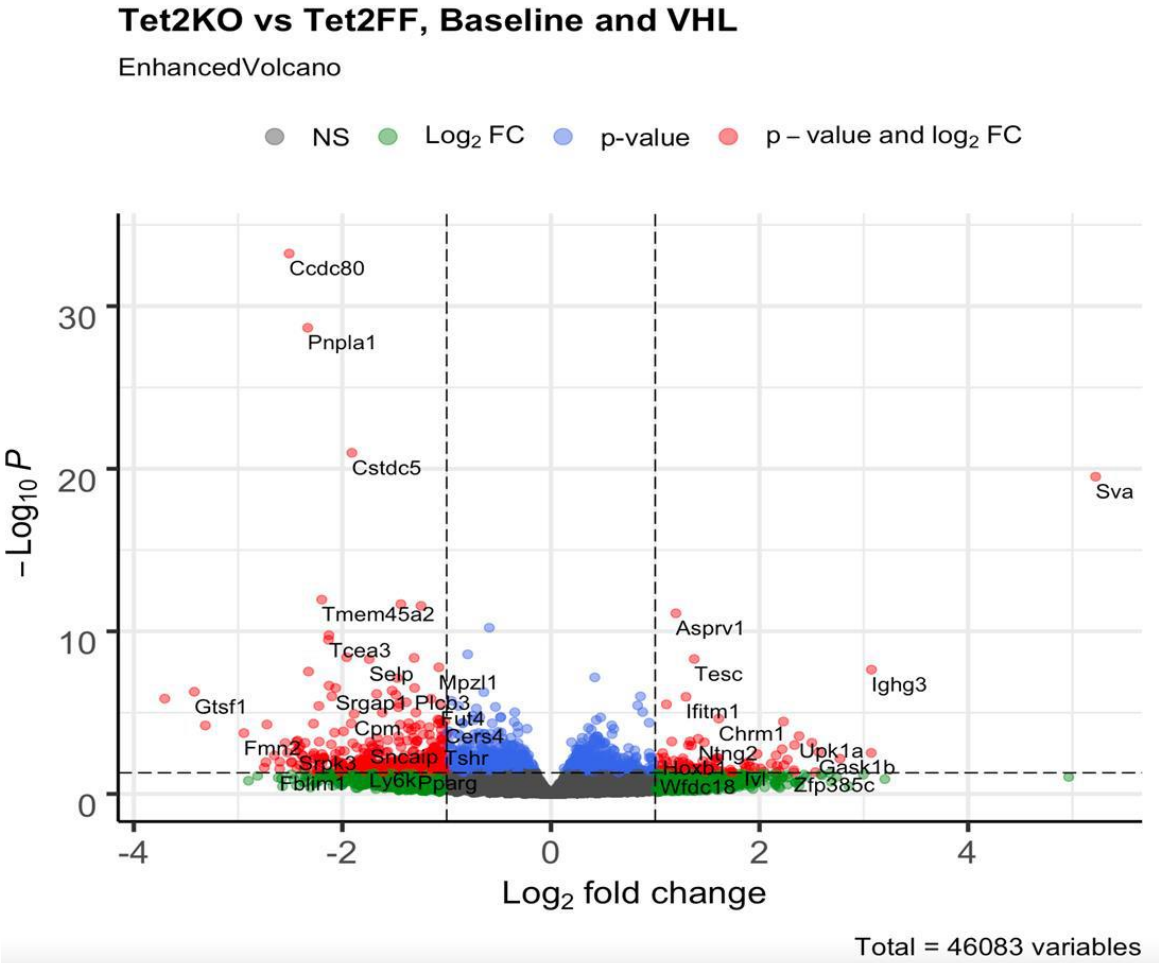

Figure S7. Volcano plot depicting differential expression of genes in *Tet2*$^{-/-}$ vs. *Tet2*$^{f/f}$ neutrophils. The LogFC cuttoff is set at Log_2=1, and the *P* value cutoff is set at *P*= 0.05. Genes on the right side of the plot are upregulated in the *Tet2*$^{-/-}$ neutrophils, while the genes on the left of the plot are downregulated in the *Tet2*$^{-/-}$ cells. Differential expression analysis was done using DESeq2 in R. Normalization was done internally through DESeq2.

Table S1. Summary of group characteristics

	No CHIP (n=16)	CHIP (n=6)
Age (yrs)	80.6 (12.1)	83.5 (12.5)
BMI (kg/m2)	26.8 (2.7)	25.4 (1.9)
Sex (n=F:M)	12:4	2:4
	% Positive (n)	
CMV positive	87.5 (14)	66.6 (4)
Smoker	6.3 (1)	16.6 (1)
**Comorbidities**		
Hypertension	18.7 (3)	16.6 (1)
Diabetes mellitus	6.2 (1)	0 (0)
Cardiovascular	0 (0)	16.6 (1)
Hyperthyroidism	6.3 (1)	0 (0)
Mood	0 (0)	16.6 (1)
Joint	18.7 (3)	33.3 (2)
GI	18.7 (3)	16.6 (1)
Respiratory	31.2 (5)	16.6 (1)
Osteoporosis	6.2 (1)	0 (0)
Other	6.2 (1)	0 (0)
Cancer history	18.7 (3)	33.3 (2)

Data showing mean (SD) unless otherwise stated. Abbreviations: *BMI* body mass index, *GI* gastrointestinal, *CMV* cytomegalovirus. Cardiovascular conditions included: hyperlipidemia. Respiratory conditions included: asthma, interstitial lung disease. GI conditions included: gastrointestinal reflux disease. Joint comorbidities included: rheumatoid arthritis and osteoarthritis. Other comorbidities included: Parkinson's disease. Comparisons of comorbidities were performed using a Fisher's exact test.

Table S2: Fluorophore-conjugated monoclonal antibodies used in flow cytometry

Cell surface marker	Fluorophore	Clone	Company	Cat No.
Mouse myeloid staining				
CD45	eF450	30-F11	invitrogen	48-0451-82
CD11b	PECy7	M1/70	invitrogen	25-0112-82
Ly6C	AF488	HK1.4	BioLegend	128022
CCR2	PE	475301	R&D systems	FAB5538P
F4/80	APC	BM8	eBioscience	17-4801-82
CD3	AF700	17A2	invitrogen	56-0032-82
CD19	AF700	eBio1D3	invitrogen	56-0193-82
NK	AF700	PK136	invitrogen	56-5941-82
Ly6G	AF700	1A8	BioLegend	127622
CX3CR1	BV650	SA011F11	BioLegend	149033
Neutrophil Maturation				
CD45	eF450	30-F11	invitrogen	48-0451-82
CD11b	APCCy7	M1/70	eBioscience	25-0112-82
Ly6C	AF488	HK1.4	BioLegend	128022
CD101	PE	Moushi101	eBioscience	12-1011-80
CCR2	BV785	SA203G11	BioLegend	150621
CX3CR1	BV650	SA011F11	BioLegend	149033
CD3	AF700	17A2	invitrogen	56-0032-82
CD19	AF700	eBio1D3	invitrogen	56-0193-82
NK	AF700	PK136	invitrogen	56-5941-82
Ly6G	PECy7	1A8	BioLegend	127617
Mouse Progenitor staining				
CD115 (M-CSF R)	AF488	AFS98	Invitrogen	53-1152-82
CD135 (Flt-3/Flk-2)	PE	A2F10	BioLegend	135305
Sca-1 (Ly6A)	PE-Dazzle 594	D7	BioLegend	108137
CD127 (IL7R)	PerCP-Cy5.5	SB/199	BioLegend	121114
Ter119	PE-Cy7	Ter119	eBioScience	25-5921-81
CD3	PE-Cy7	145-2C11	Invitrogen	25-0031-82
B220	PE-Cy7	RA3-62B	BioLegend	103222
CD117 (cKit)	BV421	2E8	BioLegend	105827
Ly6C	BV510	HK1.4	BioLegend	128033
CD16/32	BV711	93	BioLegend	101377
CD34	AF 647	HM34	BioLegend	128606
Ly6G	AF 700	1A8	BioLegend	127622
CD11b	APC-Cy7	M1/70	BD	557657
Human myeloid staining				
CD45	BV510	HI30	BioLegend	304036
CD16	PE-Cy7	CB16	eBioscience	25-0168-42
CD14	BV421	M5E2	BioLegend	301830
CCR2	PE	K036C2	BioLegend	357205
CD11b	APC	ICRF44	BD Biosciences	561015
HLA-DR	PerCPCy5.5	LN3	eBioscience	45-9956-42
CX3CR1	FITC	2A9-1	Cedarlane	D070-4
CD15	BV650	SSEA-1	BioLegend	323033
CD3	AF700	UCHT1	BD Biosciences	557943
CD56	AF700	5.1H11	BioLegend	362522
CD19	AF700	HIB19	eBioscience	56-0199-42
